# Novel Optics-Based Approaches for Cardiac Electrophysiology: A Review

**DOI:** 10.3389/fphys.2021.769586

**Published:** 2021-11-18

**Authors:** M. Caroline Müllenbroich, Allen Kelly, Corey Acker, Gil Bub, Tobias Bruegmann, Anna Di Bona, Emilia Entcheva, Cecilia Ferrantini, Peter Kohl, Stephan E. Lehnart, Marco Mongillo, Camilla Parmeggiani, Claudia Richter, Philipp Sasse, Tania Zaglia, Leonardo Sacconi, Godfrey L. Smith

**Affiliations:** ^1^School of Physics and Astronomy, University of Glasgow, Glasgow, United Kingdom; ^2^Institute of Cardiovascular and Medical Sciences, University of Glasgow, Glasgow, United Kingdom; ^3^Center for Cell Analysis and Modeling, UConn Health, Farmington, CT, United States; ^4^Department of Physiology, McGill University, Montréal, QC, Canada; ^5^Institute for Cardiovascular Physiology, University Medical Center Goettingen, Goettingen, Germany; ^6^Department of Cardiac, Thoracic, Vascular Sciences and Public Health, University of Padova, Padova, Italy; ^7^Department of Biomedical Engineering, The George Washington University, Washington, DC, United States; ^8^European Laboratory for Nonlinear Spectroscopy, Sesto Fiorentino, Italy; ^9^Institute for Experimental Cardiovascular Medicine, University Heart Center and Medical Faculty, University of Freiburg, Freiburg, Germany; ^10^Heart Research Center Göttingen, University Medical Center Göttingen, Göttingen, Germany; ^11^Department of Cardiology and Pneumology, Georg-August University Göttingen, Göttingen, Germany; ^12^Cluster of Excellence “Multiscale Bioimaging: from Molecular Machines to Networks of Excitable Cells” (MBExC), University of Göttingen, Göttingen, Germany; ^13^Department of Biomedical Sciences, University of Padova, Padova, Italy; ^14^Veneto Institute of Molecular Medicine, Padova, Italy; ^15^German Primate Center - Leibniz Institute for Primate Research, Göttingen, Germany; ^16^Institute of Physiology I, Medical Faculty, University of Bonn, Bonn, Germany; ^17^National Institute of Optics, National Research Council, Florence, Italy

**Keywords:** electrophysiology, optogenetics, heart, arrhythmia, fluorescence

## Abstract

Optical techniques for recording and manipulating cellular electrophysiology have advanced rapidly in just a few decades. These developments allow for the analysis of cardiac cellular dynamics at multiple scales while largely overcoming the drawbacks associated with the use of electrodes. The recent advent of optogenetics opens up new possibilities for regional and tissue-level electrophysiological control and hold promise for future novel clinical applications. This article, which emerged from the international NOTICE workshop in 2018^1^, reviews the state-of-the-art optical techniques used for cardiac electrophysiological research and the underlying biophysics. The design and performance of optical reporters and optogenetic actuators are reviewed along with limitations of current probes. The physics of light interaction with cardiac tissue is detailed and associated challenges with the use of optical sensors and actuators are presented. Case studies include the use of fluorescence recovery after photobleaching and super-resolution microscopy to explore the micro-structure of cardiac cells and a review of two photon and light sheet technologies applied to cardiac tissue. The emergence of cardiac optogenetics is reviewed and the current work exploring the potential clinical use of optogenetics is also described. Approaches which combine optogenetic manipulation and optical voltage measurement are discussed, in terms of platforms that allow real-time manipulation of whole heart electrophysiology in open and closed-loop systems to study optimal ways to terminate spiral arrhythmias. The design and operation of optics-based approaches that allow high-throughput cardiac electrophysiological assays is presented. Finally, emerging techniques of photo-acoustic imaging and stress sensors are described along with strategies for future development and establishment of these techniques in mainstream electrophysiological research.

## 1. Introduction

### 1.1. Electrodes vs. Light

Our current understanding of myocardial tissue electrophysiology is derived from measurements of action potential features i.e., activation, repolarization, and conduction velocity in myocardial tissue. It has been long known that the 3-dimensional (3D) structure of the tissue and regional differences in cell type composition are key determinants of electrophysiological behavior. Therefore, tools to allow interrogation and control of the electrophysiological state at various sites within myocardial tissue have high value for both research and therapy. Electrode based approaches offer the most direct way of measuring the electrical properties of the heart. However, these techniques present significant technical limitations. The gold standard for transmembrane measurements is the sharp microelectrode that gives high-resistance access to the intracellular medium at a single point to allow transmembrane voltage recording. This is in routine use in isolated tissue preparations (Hicks and Cobbe, 1991; Sicouri and Antzelevitch, 1991; Di Diego et al., 2013) but has limitations including: (i) maintenance of the delicate high-resistance intracellular access in an actively contracting tissue; (ii) the technical challenge associated with maintaining multiple simultaneous intracellular measurements to follow propagation; (iii) the difficulty in applying this technique to record transmural electrophysiology in an intact ventricular wall that in larger mammals can reach 1–2 cm thick. Despite these limitations, this technique has done much to inform scientists and clinicians of details of cardiac physiology, with work on cardiac tissue slices allowing insight into the changes in electrophysiology deep within large animal/ human myocardium (Camelliti et al., 2011). A technically simpler alternative is to use extracellular electrodes to measure potential differences between two points either on the epicardial surface, or within a solution-filled volume containing the heart. At one level this can be in the form of an electrocardiogram (ECG) used routinely for diagnostics in medicine. The most sophisticated version of this technique is body surface mapping which consists of simultaneous recordings from 300-400 points on the human chest and back. This technology, along with significant progress in solving the mathematical “inverse problem,” allows the surface body mapping to be used to create and project a 3D electrogram (Rudy, 2013) onto a 3D image of the patient's heart obtained by other clinical imaging modalities. This approach requires a very large investment in facilities and clinical expertise that currently can only be achieved in a few selected centers and cannot be easily used in experimental investigations. Furthermore, while this technique delivers 3D activation maps of the heart, a transmembrane potential signal cannot be extracted from the data. Clinically, activation mapping provides the most accurate way to diagnose conduction defects within the human heart. More routinely across clinical cardiac electrophysiology, catheter electrodes are used to detect local activation timings. These local extracellular recordings include electrode designs to provide a monophasic action potential signal using a signal from a limited depolarized region to represent the “intracellular electrode” (Franz, 1999) and needle electrodes that penetrate the myocardium to different levels to map transmural activation (Pogwizd and Corr, 1987). Cardiac stimulation in parallel with the intrinsic sinus rate activation is a necessary tool for both diagnostic and therapeutic purposes in the clinical setting and an experimental tool in research. Conventional electrodes have the same constraints as voltage measurement systems in terms of accessible surfaces of the heart chambers and the inability to stimulate discrete volumes of myocardium within the ventricle. One additional complication occurs when electrode voltage measurement and stimulation systems are used in parallel; depending on the relative positions of each, the electric fields generated by stimulating electrodes will induce transient voltages in the measurement electrodes which interfere with recording the underlying tissue response during and shortly after stimulation. As will be described in the subsequent sections of this review, many of the constraints associated with electrodes can be overcome with the use of optical probes to measure and actuate electrical signals in cardiac tissue. These optical techniques come with their own limitations, often due to the way light interacts with biological tissues. Therefore, significant sections of this review are devoted to the basic physical principles associated with light/tissue interaction.

### 1.2. Fluorescent Dyes as Indicators of Cardiac Activity

#### 1.2.1. Voltage-Sensitive Dyes

Voltage-sensitive dyes (VSD) are small fluorescent molecules that incorporate into cell membranes and report changes in transmembrane potential through changes in fluorescence. Ideally, the dye molecules should not change any “normal” functional or structural properties of the cell membrane and only integrate into the plasmalemma as opposed to intracellular membranes which are not involved in electrical activity. Furthermore, the fluorophore should ideally exhibit a high dynamic range, and respond rapidly (circa 1 ms) to any membrane potential changes.

Classic electrochromic VSD such as di-4-ANEPPS (excited in the blue-green band) have been used for decades in optical mapping experiments for detailed measurements of electrical propagation across tissues or whole organs. Since then, improved VSD have been engineered which feature increased overall brightness and voltage sensitivities through fluorination (Acker et al., 2011). Fluorination of VSD provides a tool for spectral tuning of probes, which is important for compatibility with two-photon imaging techniques (see **Box 2**) and facilitates the combination of optogenetic stimulation with optical recording (Yan et al., 2012; Crocini et al., 2016a). Another class of recently developed VSD, which relies on molecular wire photo-electron transfer (PeT), is becoming popular, thanks to their rapid response kinetics, photostability and relatively high dynamic range of fluorescence changes (20–27 %) (Miller et al., 2012). Originally developed for neuronal studies, PeT-based VSD have been used successfully in cardiac single cell and whole heart studies, proving particularly useful for deep-tissue electrophysiological measurements using two-photon imaging (Ghouri et al., 2018; Salerno et al., 2019; Klimas et al., 2020).

A common issue with dye loading is that the resulting fluorescence may display spatiotemporal variations. Uneven distribution of the probe within the tissue, heterogeneous tissue structure, non-uniform excitation light intensity, probe bleaching, motion artifacts and local changes in cell membrane structure might all contribute to time-dependent changes in detected signal. These variations then need to be removed by scaling the signal at each pixel to have the same range by calculating a ΔF/F transient.

These confounding signals can be reduced or eliminated using ratiometric approaches, where two separate signals with different responses are divided, canceling shared features while preserving or even amplifying the desired signal components. Ratiometry requires a VSD that undergoes a shift in the emission or excitation spectra depending on membrane voltage and can be employed to account for motion of cardiomyocytes (CM) and the beating heart. PeT-based VSD lack this property and therefore are unsuitable for ratiometric imaging. Care must therefore be taken in experimental design if confounding signals such as motion artifacts are thought to be a major issue. Successful use of a dye isobestic point to provide a ratiometric method that corrects for movement artifact has been successfully implemented (Bachtel et al., 2011). Ratiometric imaging has been employed for optical mapping experiments during open heart surgery (Lee et al., 2012a, 2019). Recent improvements and optimization efforts, including for ratiometric imaging, were described in a recent review article (Acker et al., 2020).

Despite remaining challenges, VSD combined with appropriate imaging methods can provide easy access to high quality voltage recordings from cell culture, tissue, or whole heart preparations with high speed and spatial resolution. Constraints continue to exist in the form of light-tissue interactions (see Box 1) which limit the precise spatio-temporal delivery of light patterns in 3D, especially at depth. This may be overcome somewhat through invasive methods, including stimulation through intramural light delivery using optrodes or implantable micro-LEDs (Byars et al., 2003; Caldwell et al., 2005; Zgierski-Johnston et al., 2020). Red-shifting of probes additionally allows one to use longer wavelengths for excitation and emission which experience less scattering and absorption in biological tissues and therefore enable extraction of signals from deeper tissue layers. Examples of such red-shifted VSD include di-4-ANBDQPQ (electrochromic; 620–660 nm excitation) and BeRST (PeT; 630 nm excitation) (Huang et al., 2015). The latter has recently been used in all-optical cardiac electrophysiology platforms for drug screening to accommodate spectrally blue-light induced optogenetic pacing and simultaneous calcium and voltage imaging (Klimas et al., 2020). For ratiometric imaging, the additional spectral bandwidth required to perform emission or excitation ratiometry may impose constraints on multi-modal acquisition. Additionally, to detect action potential features of 1 ms duration (e.g., action potential upstroke) with a fast impulse response, the sampling rate of the optical system must exceed 1 kHz. These demands require the development of sophisticated multi-modal optical imaging systems. Common issues remain across all VSD that still limit their applicability in cardiac electrophysiology; the most prominent of these being the inability to measure absolute voltages with high precision. This combined with the lack of an option for optical measurement of transmembrane current or an easy way to inject either inward or outward current optically means that an all-optical voltage clamp is not yet feasible. However, a successful implementation of an optical dynamic clamp to boost I_K1_ in human inducible pluripotent stem cell (iPSC)-derived CM has been demonstrated (Quach et al., 2018).

Box 1 **Light penetration in tissue**

**Physical Basis for Light Scattering in Tissue**

Light of a given wavelength can be described as an electromagnetic wave in which the electric and magnetic components oscillate at a particular frequency. It is the vibration of the electric component of light (typically at ≈10^15^ Hz for red light) which is responsible for scattering: the wave's vibrational energy causes valence electrons in surrounding media to vibrate transiently. The energy absorbed by the electron is quickly released in its entirety again to generate a spherical wavelet of the same frequency (energy conserving elastic scattering). The repetitive absorption and re-emission of energy, typically in the order of femtoseconds for visible light, causes a cumulative delay, which is directly proportional to the number of molecules and hence of electrons the light can interact with per unit volume. The degree to which light slows while propagating through a medium is quantified by the refractive index of the media, which reports the ratio of speed of light in a vacuum (the only place where zero scattering occurs) divided by the speed of light in the medium. Apart from molecule density, the susceptibility of electrons to interact with light also affects the refractive index, which explains why lipid-rich structures such as cell membranes have a higher refractive index than surrounding water (1.45 vs. 1.33) even though they have a lower density (Richardson and Lichtman, 2015).

**Scattering**

Scattering occurs everywhere light enters a medium, even in transparent media like air, glass or water. In contrast to opaque media, transparent media have two important properties. Firstly, the density of scatterers is so high that many scatterers exist over distances much smaller than the wavelength of light. Secondly, the scatterers are homogeneously distributed which means that lateral scattering is effectively canceled out by destructive interference of secondary wavelets and no light propagates perpendicular to the impinging light wave. In the forward direction, scatterers are activated sequentially and their excitations sum constructively, leading to attenuation-free propagation of light. When a medium is not homogeneous but instead contains structures whose size is comparable with the wavelength of the light (300–700 nm for visible light), strong Rayleigh scattering occurs. The magnitude of Rayleigh scattering is inversely proportional to the fourth power of the wavelength which means that longer wavelengths are scattered less than shorter wavelengths. In biological tissue heterogeneous structures include cellular constituents, such as ribosomes, nuclei, nucleoli, mitochondria, lipid droplets, membranes, myelin, cytoskeletal components, and extracellular matrix constituents such as collagen and elastin. These scatterers are inhomogenously distributed and give rise to substantial lateral scattering which no longer cancels out entirely due to destructive interference. Multiple scattering over a sufficient tissue thickness leads to tissue opacity.

**Light Penetration in Tissue**

Light can be described as an electromagnetic wave characterized by its wavelength, its power (watts *W* = Joule/second) and its irradiance (intensity, *W*/*m*^2^). Only in homogeneous media does light propagate along a straight line. In this case, the energy of light is mostly lost through absorption of the medium and is converted to thermal energy, fluorescence or photobiochemical reactions. In elastic scattering events, the energy of the light is not lost but the direction of light propagation changes. Consequently, irradiance decreases in the direction of propagation in the measure that scattering causes lateral spreading of the light.

The intensity of light passing through a scattering and reflecting sample is given by

(1) $$\begin{array}{r} I_{\lambda }\left(d\right)=\left(1-R\right)I_{0}\exp\left(-\epsilon_{\lambda }d\right) \end{array}$$

where *d* is the distance traveled in the medium, *R* is the reflectance, and ϵ_λ_ is the extinction coefficient which is the sum of the medium's absorption and scattering coefficients (Deng et al., 2014). Using this framework, light scattering can be quantified by a single number; the penetration depth *l* which describes the depth at which light decays to 1/*e* (about one third) of its incident intensity *I*_0_ and is given by the inverse of the extinction coefficient of the medium: *l* = 1/ϵ_λ_.

**Absorption**

Hemoglobin, myoglobin, and melanin are the primary molecules responsible for visible light absorption in heart tissue. The “near-infrared window” is a section of the electromagnetic spectrum in which biological tissue only weakly absorbs, usually using light with wavelengths of 650–1.350 nm for example in near-infrared (IR) fluorophore excitation or with two-photon microscopy (see Box 2).

#### 1.2.2. Calcium Sensitive Dyes

Due to the fundamental importance of Ca^2+^ in the excitation contraction coupling process in CM, we briefly touch upon calcium (Ca^2+^) imaging which optically quantifies $\text{C}{\text{a}}_{\text{i}}^{2+}$ transients. Still extensively used since their development in the 1980s, fluorescent probes developed in the Tsien lab combine a Ca^2+^ chelator with a fluorophore, for example fluorescein, stilbene or rhodamine, within the same molecule. A variety of probes are now available which display a range of quantum efficiencies, photobleaching stabilities, sensitivities, and selectivities for Ca^2+^, as well as differences in excitation spectra and temporal resolution. Some probes shift in emission wavelength upon Ca^2+^ binding to enable ratiometric quantitative measurements of the intracellular free Ca^2+^ concentration (Fura-2, Grynkiewicz et al., 1985), while others are non-ratiometric (Rhod-2). A recent review on ratiometric and non-ratiometric $\text{C}{\text{a}}_{\text{i}}^{2+}$ probes including probes for imaging of sarcoplasmic reticulum Ca^2+^ and probes compatible with potentiometric voltage dyes for multi-modal optical mapping is provided elsewhere (Jaimes III et al., 2016). As with VSD, careful consideration must be given to Ca^2+^ probe compartmentalization and how it could be influenced by probe concentration and loading conditions, especially duration and temperature. Measuring intracellular Ca^2+^ also offers a set of considerations which strongly influence the choice of indicator. Molecule binding affinity determines the Ca^2+^ concentration range within which the indicator is useful. High affinity indicators such as Fluo-3 and Fura-2 are appropriate for cardiac cytosolic Ca^2+^ measurements. However, many intracellular compartments contain much higher concentrations (such as the sarcoplasmic reticulum where Ca^2+^ levels approach 1 mM range) and require low affinity indicators, for example Fluo-5N (Wu and Bers, 2006). It is also important to remember that indicators themselves act as Ca^2+^ ion buffers. Therefore, minimizing the influence of the indicator on physiological processes requires a trade-off between keeping intracellular dye concentration as low as possible while maintaining good signal-to-noise ratio (Paredes et al., 2008).

### 1.3. Optogenetics: From Genetically Expressed Indicators to Light-Sensitive Ion Channels

Optogenetics, in its broadest sense, describes a set of tools which perform complementary functions: light-driven actuators impose targeted perturbations of either electrophysiological signals (Nagel et al., 2002) or activate intracellular signaling pathways e.g., G-protein coupled receptors (Makowka et al., 2019), while light emitting reporters reveal them (Miesenböck, 2009). This powerful combination of active interrogation and passive observation affords one the ability to infer spatiotemporal dynamics of complex systems that cannot be easily elucidated from the activity of their individual components (Entcheva and Bub, 2016). The genes coding for optogenetic proteins, be they light-emitting reporters or light-driven actuators, are encoded in DNA vectors and introduced under the control of a (potentially cell type-specific) promoter into the genome of the host organism. This causes selected cells to produce the optogenetic proteins with the cell's endogenous synthesizing machinery. Using the cell itself to express the reporter (or actuator) overcomes some of the aforementioned limitations of intracellular loading associated with exogenous probes. The combination of cell-selectivity in gene expression and wavelength-specificity of protein activation, provide the outstanding scientific value of optogenetics. The possibility to spectrally combine optical actuators and reporters allows for completely contact-free all-optical interrogation and control. Recent comprehensive reviews details cardiac applications of optogenetics over the last decade (Koopman et al., 2017; Entcheva and Kay, 2021).

Optogenetic reporters translate CM electrophysiological signals into optical signals. Virtually all genetically-encoded Ca^2+^ indicators (GECI, reviewed in Kotlikoff, 2007; Whitaker, 2010), are derivatives of the green fluorescent protein (GFP). For example, G-CaMP modulates its output signal through Ca^2+^-dependent conformational changes that allow or restrict solvent access to the chromophore, which causes increased brightness with Ca^2+^ binding (Chen et al., 2013).

Genetically-encoded voltage indicators (GEVI) report changes in membrane potential from individual cells or populations using voltage-sensitive fluorescent proteins. Current GEVI are limited by the brightness and photostability of fluorescent proteins. The electric field decreases exponentially with distance from the cell membrane, which means the available volume for voltage indicators is restricted to the cell membrane, i.e., a very small fraction of the total cell volume. Additionally, because membrane potential changes can occur over very short timescales, only a few photons are emitted from a restricted volume over a restricted time making their detection statistically difficult. Expressing more reporter molecules bears the risk of observation interfering with the very quantity of interest, electrical excitability, because the mobile charges of voltage probes sensing the cellular membrane potential add a capacitive load to the cell membrane. Whether this increased membrane capacitance influences action potential characteristics is unclear, and seems to depend on the target system, the specific GEVI being used and its overall expression levels (Yang and St-Pierre, 2016). The most commonly used GEVI in cardiac *in vitro* applications so far is ArcLight (Jin et al., 2012), a bright GEVI with relatively slow kinetics, limiting its ability to capture detailed action potential morphology. A new chemigenetic GEVI, Voltron, combines the specificity of genetically encoded reagents with the superior photophysics of synthetic rhodamine dyes like the Janelia Fluor instead of protein-based fluorophores. In a hybrid approach, a genetically expressed scaffold, composed of a voltage-sensitive microbial rhodopsin domain with a dye-capture protein domain, irreversibly binds the synthetic dye (Abdelfattah et al., 2019). Changes in membrane potential alter the absorption spectrum of the rhodopsin domain in Voltron which in turn reversibly modulates the degree of fluorescence quenching of the dye through Förster resonance energy transfer (FRET), yielding a GEVI with increased photon output.

In addition to being used as reporters, optogenetics also includes a suite of genetically-expressed photosensitive actuators which alter the ionic flux through cellular membranes in response to light of specific wavelengths (Nagel et al., 2002). For example, upon illumination with 465 nm, Channelrhodopsin-2 (ChR2) (Nagel et al., 2003; Boyden et al., 2005) a light-sensitive cation channel opens and depolarizes the cell, which can be used to elicit action potentials in excitable cells such as neurons and CM. These channels have been extensively color-tuned (Prigge et al., 2012) and ion pumps expand the toolset to bi-directional control of transmembrane voltage. For example, light-activated potassium channels have been used to inhibit activation by hyperpolarizing the cell (Sierra et al., 2018). Optogenetic actuation allows one to conduct light stimulation or inhibition of cells that do not normally respond to light, supporting the investigation of causal relationships within networks of cells. An exciting development in optogenetics is the possibility of optically controlling exogenous gene expression itself (De Mena et al., 2018). Further manipulation of natural photo switches has expanded the toolkit from light-activated membrane channels to non-channel proteins designed to induce protein-protein interactions (Deisseroth, 2011). These new interaction tools include light-activated enzymes and transcription factors which permit expression of genes in locally defined tissue regions. An important consideration when using optical approaches is the amount of light required to either create sufficient fluorescence signal to be detected or to activate a light-driven protein function. This value is usually a balance between the need to maximize excitation/ photo-conversion and need to minimize photo-bleaching and cellular photo-damage. Table 1 indicates the light irradiance needed at the sample/ tissue/ cell used in the study of cardiac tissue using a range of light sources and optics. For single photon applications approximately 10–100 mW μm-2 is commonly used, while two-photon applications require approximately 10,000 higher power but at approximately double the wavelength.

**Table 1:** Values in this table indicate the approximate irradiance required from different light sources at the sample to image Ca^2+^ or activate ChR2.

**Light sources**	**Wavelength (typical) (nm)**	**Illumination optics (typical)**	**Irradiance**
Light emitting diodes/ Xe lamps	400–700	Macro/microscope objective	10 mW mm-2 Funken et al., 2019, 2020
Continuous wave laser (single photon)	400–700	Microscope (1 μm × 1 μm spot)	5–10 μW μm-2 Centonze and White, 1998
Ultrashort pulsed laser (two photon)	700–1100	Microscope (1 μm × 1 μm spot)	5–10 mW μm-2 Bush et al., 2007

*The wavelengths are chosen to represent common voltage/ Ca^2+^ fluorescent indicators and optogenetic applications*.

## 2. Multimodal Imaging of Intracellular Ca^2+^ and Membrane Potential

Cardiac excitation-contraction coupling is governed by the complex and dynamic interplay between membrane ion fluxes and intracellular Ca^2+^ handling processes and manifested in the action potential and intracellular Ca^2+^ transient, respectively. Multimodal acquisitions, i.e., the simultaneous monitoring of calcium and voltage at high spatiotemporal resolution, are important because they provide insight into excitation contraction coupling and arrhythmia mechanism (Salama and Hwang, 2009). The prevailing technique of concurrent tracking of fluorescent reporters of either membrane voltage or cytoplasmic Ca^2+^ is optical mapping (Jaimes III et al., 2016).

### 2.1. Optical Mapping

In optical mapping the most basic fluorescent microscope, a wide-field microscope (or macroscope for large field of view), is employed to flood-illuminate the sample and detect the fluorescence of indicators across the entire field of view. For the heart, in particular Langendorff setups, different geometries can be used that can be based on photodiode arrays (Efimov et al., 1994; Qu et al., 2007) or camera(s) with suitable lenses. This can be single-view (Lee et al., 2011), multiple-camera / multiple-view (Cathey et al., 2019) or single-camera/ multiple-view (Lee et al., 2012b). The wide-field illumination and detection scheme has the advantage of being fast and simple, and it can obtain both topographical and dynamic information. Usually, mercury lamps or LEDs are used for fluorescence excitation and if functional data are to be acquired, a high-speed imaging system is needed to capture and visualize small changes in fluorescence intensity. Considering the minimum sampling frequency necessary, according to the Nyquist sampling theorem, to achieve a temporal resolution suitable for tracking action potential propagation (*ms*) the camera employed in optical mapping must operate at a minimum frame rate of ~2*kHz*. Frame rate requirements for Ca^2+^ imaging, where Ca^2+^ release from the sarcoplasmic reticulum is the fastest part of the transient and time to peak is in the order of ~ 10–30 ms, are more relaxed. The high costs associated with setups capable of high frame rates can be circumvented by “temporal pixel multiplexing” techniques that embed temporal information in still images to enhance the temporal resolution of imaging methods (Bub et al., 2010). Further requirements for the camera, typically a charge coupled device (CCD) or scientific complementary metal-oxide semiconductor (sCMOS) camera, are very low read-out noise, high quantum efficiency and high well depth to capture the small fluorescence variations associated with action potential or Ca^2+^ release with enough dynamic range and signal-to-noise ratio. Multimodal acquisition can be achieved in several ways. Certain combinations of Ca^2+^ indicators and VSD can be excited by a single light source due to overlapping excitation spectra but distinct emission bands, thereby minimizing signal cross talk (for example the Ca^2+^ indicator Rhod-2 and VSD RH-237, Salama and Hwang, 2009). Separation of the emitted fluorescence signal into different detectors (or different areas of the same sensor) can be achieved with dichroic mirrors and emission filters (Yan et al., 2012). Multiplexing can also occur by excitation, albeit at a reduced temporal resolution, by turning different LEDs on and off sequentially during synchronized image acquisition to excite spectrally-separable voltage and calcium probes during synchronized image acquisition (Lee et al., 2011; Klimas et al., 2020). Low-magnification cardiac optical mapping *in vitro* presents higher demands on the imaging systems compared to whole heart imaging due to the very thin layer of cells generating a signal (Entcheva and Bien, 2006). Hence the need for high- numerical aperture (NA) lenses borrowed from photography, more sensitive detectors and better fluorescent reporters to map voltage and Ca^2+^ excitation in human induced pluripotent stem-cell-derived CM, for example.

One issue that has featured in the majority of optical mapping studies is the requirement that the underlying epicardium does not move substantially during the imaging process over the contraction/relaxation cycle. In early studies, this was achieved by gently pressing the right ventricle/ left ventricle (LV/RV) surface against an optical surface during image capture (Kanai and Salama, 1995). This procedure requires care to prevent the development of ischaemic areas when physically restraining the heart. More commonly, contraction related movement is minimized by chemical electro-mechanical uncouplers such as the myosin inhibitors butanedione monoxime (Kettlewell et al., 2004) or blebbistatin (Fedorov et al., 2007). These compounds block contraction by direct inhibition of the myofilaments, thus limiting motion artifacts whilst preserving electrophysiological function. Though there is published evidence for direct effects of most of the common uncouplers on electrophysiology (Kettlewell et al., 2004; Brack et al., 2013; Kappadan et al., 2020), the magnitude and importance of this limitations is debated. Derivatives of blebbistatin, i.e., para-nitro blebbistatin (Képiró et al., 2014) and para-amino blebbistatin (Várkuti et al., 2016) have chemical features that facilitate their use as uncouplers but similarly require rigorous testing for non-specific effects on cardiac electrophysiology.

In addition to the potential direct effects of chemical uncouplers on cardiac electrophysiology there is also the physiological influence of muscle length and contraction on electrophysiology via a series of mechano-electric feedback (MEF) systems (Quinn and Kohl, 2021). This issue can be circumvented by the application of processing algorithms to the moving isolated heart to track epicardial regions during contraction, thereby minimizing movement artifact (Christoph and Luther, 2018). These approaches are not system-specific and their use is restricted to the few labs able to support the complex post-acquisition analysis. The outcome of these approaches is not just the electrical map of the imaged surface, but the mechanical behavior of the myocardium, by itself an important physiological feature and one which informs the study of phenomena associated with MEF.

Myocardial motion alone can be used to infer activation timings and patterns in the absence of probes to monitor electrical activity (Christoph et al., 2018). When applied to the whole heart during the cardiac cycle, this approach has the complication of interpretation as movement depends on a number of factors including preload and afterload. With this caveat, computationally streamlined image analysis techniques have been developed to track motion of contracting tissue (Burton et al., 2015; Sala et al., 2018). Among other advantages, dye-free imaging works at all wavelengths, which simplifies its integration with spectrally-restricted opsins used for actuation (Burton et al., 2015) and in mechanically similar muscle systems such as cultured CM monolayers these techniques can map patterns of contraction over long periods of time. Furthermore, recently developed random access parallel imaging, inspired by a Newtonian telescope-based addressing of samples without moving parts, demonstrated high-throughput dye-free imaging in 96-well format (Ashraf et al., 2021). An important consideration when analyzing optical mapping data from thick tissue or whole heart preparations is that this technique lacks optical sectioning. Since illuminating light penetrates several millimeters into highly scattering cardiac tissue, the resultant electrophysiological signals arise from a volume below the tissue surface. As a result, signal features such as transient upstroke morphology and duration are prolonged (Girouard et al., 1996), particularly in optical action potentials. This also has implications for multimodal imaging setups utilizing distinct wavelength excitation light sources, since the longer wavelength light will penetrate deeper, exciting a larger volume of tissue. These features appear at first disadvantageous, however the fact that optical action potentials carry information about the electrophysiology of a tissue volume has been used to reveal subepicardial fiber orientation based on action potential upstroke shape (Hyatt et al., 2005). Dual excitation of spectrally separated VSD has also been used successfully to reveal unique information about transmural heterogeneities and endocardial electrophysiology in rat hearts (Walton et al., 2010). Looking to the future, with the emergence of low-cost high-speed cameras, volumetric optical mapping in small-size hearts has been shown to be possible with multi-plane multi-camera parallel imaging (Sacconi et al., 2020).

### 2.2. Two-Photon Microscopy for Deep Tissue Access

While optical mapping has proven to be a powerful and versatile tool, in its classic implementation, lack of depth discrimination limits the degree of information on transmural electrophysiology. The increasing need to investigate biological specimens in 3D has driven the development of optical techniques capable of volumetric imaging. As opposed to physical sectioning, these techniques, most notably confocal, two-photon (TPM) and light-sheet microscopy, use optical sectioning to obtain depth-specific information.

In confocal microscopy the sample is sequentially illuminated by a diffraction limited spot, typically employing single photon excitation, and the signal from this spot is registered using a point detector masked by a small pinhole. Similar to TPM, confocal microscopy is a serial sampling instrument which uses the same objective for excitation and detection (epi-configuration). The pinhole gives confocal microscopy its optical sectioning capability because it prevents or at least severely attenuates the detection of light emitted from areas outside of the focal volume. The pinhole is placed in an image plane and typically has a diameter of 1-2 Airy units. The image of the pinhole in the object plane restricts the volume from which photons will be collected, thereby increasing image contrast.

In confocal and wide-field microscopy, contrast is generated from light-matter interactions in which the elementary process (absorption, scattering, etc.) only involves a single photon and which therefore depends linearly on the incident light intensity. Non-linear microscopy methods, such as TPM (Denk et al., 1990) rely on higher-order light matter interactions involving two or more photons for contrast generation. This fundamental difference is the source of numerous new imaging properties. TPM is especially well-suited for deep tissue imaging as it supports imaging scattering samples non-invasively with sub-micrometer resolution in 3D. Moreover, due to the excitation process, which results from the simultaneous absorption of two photons, there are a number of unique advantages such as reduced specimen photodamage and enhanced penetration depth.

### 2.3. Optical Sectioning Microscopy in the Intact Heart

Optical sectioning techniques like confocal and two-photon microscopy enable one to look beneath the sample surface to resolve cellular processes and structures within discrete volumes of tissue. A wealth of information can therefore be gained from measurement of cardiac cellular electrophysiology deep beneath the epicardium of the intact heart. Cardiac TPM allows for both *in vivo* and *ex vivo* cellular and cell layer-scale electrophysiological and intracellular Ca^2+^ measurements across the myocardial wall (Rubart et al., 2003; Kelly et al., 2013, 2018; Ghouri et al., 2018). Cardiac TPM has been critical in defining the importance of structural features for transmural conduction, both in healthy tissue and in models of reduced tissue excitability (Kelly et al., 2013, 2018) including confirmation of heterocellular electrical coupling of cardiac myocytes and fibroblasts (Rubart et al., 2018), previously established by optical mapping (Quinn et al., 2016). This is aided significantly by the ability to perform simultaneous two-photon fluorescence and second harmonic generation microscopy, enabling label-free imaging and quantification of collagen in live tissue, an important marker of pathological remodeling in cardiac disease (Martin et al., 2013). For *in vivo* TPM, the motion of the heart becomes a significant technical challenge which cannot be overcome with chemical uncouplers. However, several publications have detailed methodological considerations to allow this type of imaging (Lee et al., 2012c; Vinegoni et al., 2015), which has aided in better understanding of cardiac immune cell motility and trafficking (Kreisel et al., 2010; Li et al., 2013). Here we describe several avenues of cardiac electrophysiological research which continue to benefit from developments in TPM.

#### 2.3.1. Transmural Imaging

Electrical propagation under normal circumstances adopts a significant transmural axis, from the endocardium toward the epicardial surface. Within the intramural space, this electrical wavefront encounters an array of physical discontinuities; blood vessels, extracellular clefts and regions of heterogeneous fibrotic distribution. The laminar arrangement of CM within the ventricle shift and rotate at right angles to each other, sometimes abruptly (within 2–3 cell layers of thickness) (Hooks et al., 2007). These factors have a measurable impact on electrophysiology, even in healthy hearts. In combination with optical mapping, *ex vivo* whole heart recordings yield a wealth of information about ventricular electrophysiology across a wide region, which can then be examined at the cellular scale within the transmural wall, affording greater detail about the influence of microscopic myocardial wall tissue features (cell orientation, cell distribution, microarchitecture, including the influence of scar tissue in disease) on electrophysiology and transmural conduction. As with optical mapping, electrophysiology can be tracked with rapid scanning TPM in conjunction with VSD such as di-4-ANEPPS, albeit over a restricted field of view using a high-NA objective. The optical sectioning power of TPM however, allows for action potential characteristics at a given transmural depth to be faithfully captured, with 2 kHz scanning rates capable of resolving action potential rise time characteristics in multiple small animal species with similar fidelity to sharp microelectrodes (Ghouri et al., 2015). Serial scanning necessitates slow volumetric rates, which requires electrical stimulation of the heart to generate a repeated sequence of activation, a common approach in isolated perfused heart experiments. This carries the added caveat however that beat-to-beat variability, an important property of whole heart electrophysiology, cannot be examined. Thus, an average profile of transmural electrical conduction can be constructed from sequential volumetric measurements using the electrical stimulus as a timing reference. This approach revealed that surface boundary conditions measurably increase action potential upstroke velocity as the wavefront approaches the epicardial surface transmurally, demonstrating the strong electrotonic load influence on CM action potential properties (Kelly et al., 2013). This technique was also key in describing conduction abnormality in a model of Brugada syndrome, an inherited arrhythmic disorder often characterized by slow conduction, right ventricular arrhythmias and higher risk of sudden death. Combining TPM with computational simulations, it was found that the less compact structural arrangement of the right ventricle made it uniquely susceptible to conduction slowing when myocyte excitability is reduced, particularly at higher heart rates (Kelly et al., 2018).

#### 2.3.2. Electrical Coupling of Implanted iPSC-Derived CM Grafts After MI

The intrinsic regenerative capacity of the heart is limited; estimates place cell turnover in adult humans at 0.5–1 % *per annum* (Bergmann et al., 2009). As a result, ischemic insults (myocardial infarction) activate inflammation-mediated signaling cascades which replace necrotic tissue with a vascularized infarct scar, reducing the overall contractile capacity of the heart and forming a substrate for ventricular arrhythmias. One of the promising repair strategies to emerge in recent years involves implantation of engineered heart tissue (EHT) constructs, grown from iPSC-CM, directly over the infarct to restore function. This has met with some success in both small (Matsuo et al., 2015; Pecha et al., 2019) and large animal models (Gao et al., 2017), but questions remain over the extent of EHT coupling to native myocardium, as well as the long-term survival of the originally-implanted cells. Addressing these questions has generated a number of technical challenges that TPM can help overcome (Weinberger et al., 2016). Electrophysiological assessment via optical mapping of voltage sensitive dyes alone struggles to fully distinguish between electrical activity of the EHT and normal myocardium. It has been known for some time that fluorescent “bleed-through” from surviving endocardial tissue (or, if the infarct is thin enough, septal tissue) will contaminate the optical map of the infarct with implanted EHT, giving the appearance of relatively healthy looking action potentials from largely-free scar. Gene-silencing of G-CaMP indicators expressed in the EHT was also observed to occur when hearts were explanted 4 weeks post-surgery. TPM helped to overcome these setbacks by allowing delineation of EHT and infarct electrophysiology through serial optical sectioning, demonstrating that electrical coupling of EHT and native myocardium can occur, albeit with a relatively low success rate (Weinberger et al., 2016). While the above patches were developed with the aim of aiding contractile function, another exciting application of engineered tissue is to serve as electrical conduction tracts. This has been shown to efficiently bridge atrio-ventricular conduction in a murine model of atrio-ventricular node failure (Cingolani et al., 2014), and shows that electrical integration of engineered tissue constructs can indeed be achieved with potentially far-reaching consequences for translation (Kohl, 2014). In any case, the further development of EHT-based organ repair is uniquely poised to benefit from the discriminating power that TPM optical sectioning provides.

#### 2.3.3. Origin of the Electrical Conduction Through Myocardial Infarct Scar

Questions remain over electrical interaction between the infarct scar and healthy myocardium. Surviving islands of myocardium and remnant electrical activity within the infarct are well-documented (Walker et al., 2007), with myofibroblasts implicated in permitting electrical propagation between the border zone and surviving myocardium within the scar itself (Quinn et al., 2016). Several studies using TPM have sought to elucidate the mechanism of infarct conduction. Combined optical mapping-TPM systems and dual voltage and Ca^2+^ labeling has been used to distinguish between myocyte and non-myocyte electrical propagation within the infarct zone in rat hearts, using the absence of an intracellular Ca^2+^ transient as an indicator of non-myocyte electrotonic interaction (Ghouri et al., 2018). A similar analysis of the peri-infarct zone in mice expressing CM-specific GFP, identified non-myocyte electrical activity attributed to myofibroblasts exhibiting markedly slowed action potential upstrokes (Rubart et al., 2018). Engraftment of Connexin-43 (Cx43) expressing non-myocytes (Roell et al., 2007), or over-expression of Cx43 in murine infarct tissue (Roell et al., 2018), aided trans-scar conduction, implicating heterocellular contributions to electrical propagation in the heart. A thorough investigation of myocyte-fibroblast interaction within mouse infarct using a similar CM-specific labeling approach is currently being undertaken.

### 2.4. Random Access Multiphoton Microscopy in the T-Tubular System

**Figure 1:**
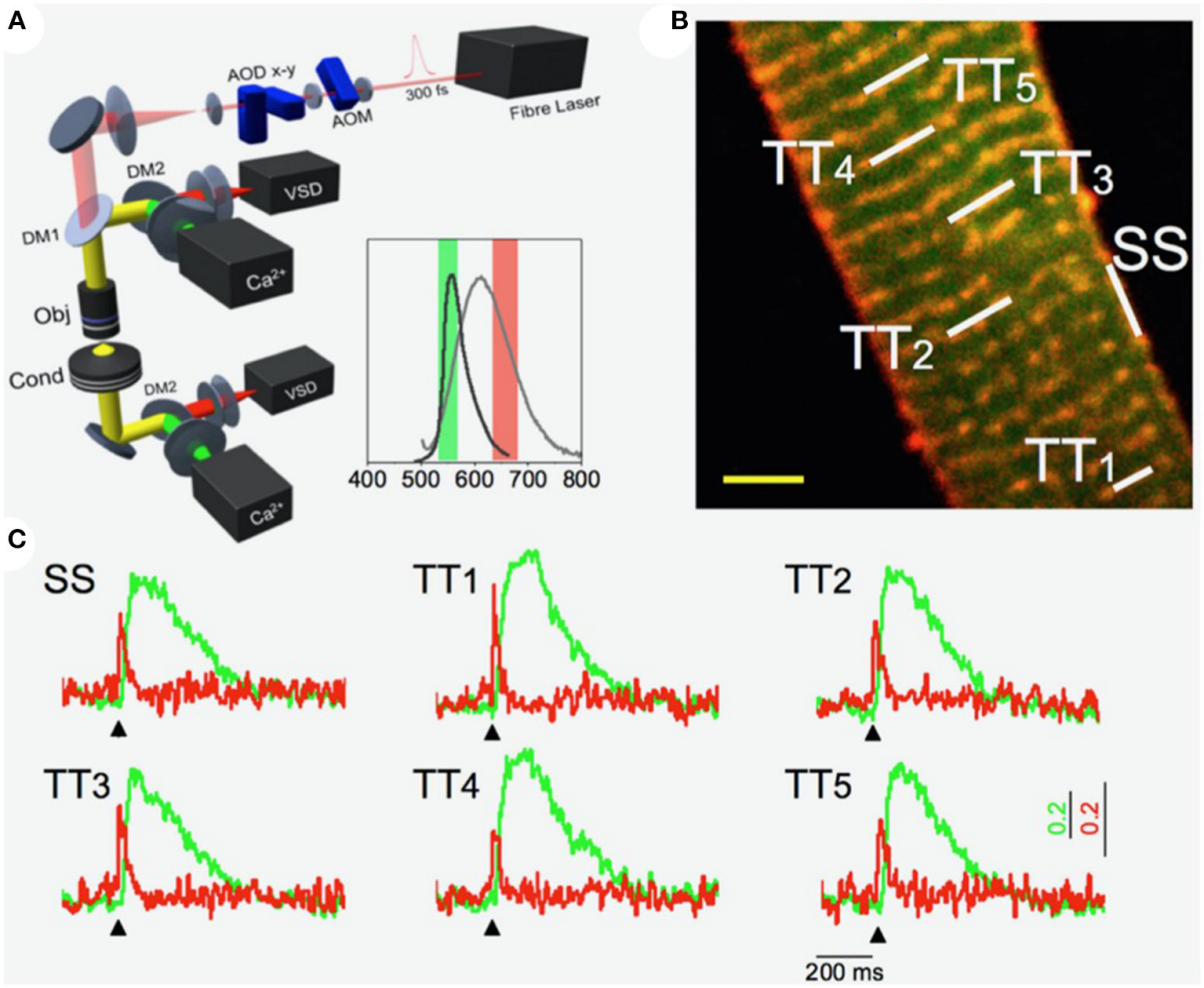
Multi-site voltage and Ca^2+^ recording. **(A)** Scheme of the Random Access Multi-Photon (RAMP) microscope based on two orthogonally-mounted acousto-optical deflectors (AODs -x and -y) for laser scanning. Inset shows the emission spectra of the Ca^2+^ indicator (in dark gray) and voltage sensitive dye (in light gray) together with their fluorescence filter acquisition band. **(B)** Image of a stained rat ventricular cardiac cell: di-4-ANE(F)PPTEA in red and GFP-certified Fluoforte in green. Scale bar: 5 μm **(C)** Real time fluorescence traces collected from the scanned sites indicated in white in **(B)**: surface sarcolemma (SS) and five T-tubules (TTi). Membrane voltage is shown in red and Ca^2+^ in green. Reproduced and modified from Crocini et al. (2014).

Random access multiphoton microscopy (RAMP) (Sacconi et al., 2012) based on acousto-optic deflectors (AODs) was recently used in combination with novel VSD with improved photostability (Yan et al., 2012) to measure T-tubular action potentials at multiple sites with high spatio-temporal resolution. The authors observed that a tight electrical coupling between distinct membrane domains (i.e., surface sarcolemma and T-tubules) is guaranteed only in healthy cells. Conversely, experiments on disease models showed that action potential propagation in the structurally remodeled T-tubular system can fail and local spontaneous depolarizations can occur in the electrically disconnected T-tubules (Sacconi et al., 2012). A simultaneous recording of the action potential and local Ca^2+^ release was used to dissect the consequences of electrical defects on intracellular Ca^2+^ dynamics. Specifically, the authors implemented a double staining approach, combining a Ca^2+^ reporter (FluoForte GFP-certified) with a fluorinated VSD. Two-photon excitation features were exploited to simultaneously excite both dyes, while band-pass filters were used to select the two distinct spectral ranges of the overlapping fluorescence spectra. A spectral unmixing procedure was applied to properly uncouple voltage and Ca^2+^ signals. Employing this approach in combination with the random access modality, the authors were able to optically record action potentials in several T-tubules and, simultaneously, the corresponding local Ca^2+^ transients (Crocini et al., 2014). Figure 1 shows an example of an optical recording of voltage and Ca^2+^ release in six different positions across the sarcolemma. The signal-to-noise ratio of the proposed method was sufficient to detect the presence of an action potential and to assess the temporal features of Ca^2+^ dynamic in single shot recordings, without averaging sequential trials. The utility of the system in detecting electrical activity and local Ca^2+^ release simultaneously in multiple sites was used to solve unanswered questions about the spatio-temporal relationship between sub-cellular alterations of Ca^2+^ release and T-tubular electrical and structural defects in several cardiac diseases including ischemic heart failure (Crocini et al., 2014), spontaneously hypertensive rats in overt heart failure (Scardigli et al., 2018a), and hypertrophic cardiomyopathy (Crocini et al., 2016b).

### 2.5. Enhancing the Utility of Two-Photon Optical Sectioning Microscopy for Cardiac Applications

Non-linear imaging (Box 2) is uniquely applicable to transmural cardiac studies and combined with several adjacent technologies, including optical mapping and emerging optogenetic tools, provides a powerful and versatile analytical tool for cardiac electrophysiology. Despite this, significant challenges remain to prevent its widespread adoption which are intrinsic both to the technology itself and cardiac muscle in particular. Understanding these limitations and available options for overcoming them is critical for informing the next generation of 3D cardiac imaging studies based on TPM.

Box 2 **Two-photon microscopy**

**Physical Background**

In conventional fluorescence spectroscopy, a fluorescent molecule is excited by absorption of one photon whose energy corresponds to the energy gap between the ground state and the excited state of the molecule. However, the same excitation is also possible by the near-simultaneous absorption, i.e., within 1e-16 s, of two photons of approximately double the wavelength via a short-lived, virtual intermediate state. The combined energy of the two long-wavelength, low-energy photons produce an excitation equivalent to the absorption of a single photon possessing twice the energy. The emitted fluorescence generated by the simultaneous absorption of two photons is indistinguishable to single photon excitation fluorescence emission. The simultaneous absorption of two photons is the origin of the quadratic dependence on the light intensity: in TPM, doubling the excitation intensity produces four times the fluorescence. This non-linear dependence of two-photon absorption is the basis for the intrinsic localization of fluorescence generation and therefore the optical sectioning capability, i.e., the imaging of specific planes within the sample without the need for physical slicing.

**Requirements**

Like most non-linear processes, the excitation probability in two-photon excitation fluorescence is extremely low but can be optimized to generate enough signal by concentrating the excitation light in space and time. Spatial compression is achieved by tight focusing with high-NA objective lenses. This increases the local intensity at the focal point and with it the probability for two-photon excitation. Away from the 3D focal spot the probability for simultaneous absorption drops off drastically such that virtually no fluorescence is generated outside the focal volume (Zipfel et al., 2003). Scattered excitation light is too dilute to excite by two-photon absorption and too long in wavelength to be absorbed otherwise. Owing to the low two-photon absorption cross-section inherent to non-linear processes, a useful fluorescence signal can only be achieved with ultrashort pulses which compress excitation intensity in the time domain. Ultrafast pulsed lasers concentrate photons into very short (pulse width τ), high peak intensity pulses separated by e-8 intervals (repetition rate *R*), keeping the average power relatively low. This increases the signal by a factor of 1/(τ*R)^*n*−1^ compared to continuous-wave illumination, where *n* is the number of photons involved in the elementary process (Helmchen and Denk, 2005). At present, the most commonly used lasers in TPM setups are mode-locked titanium sapphire (Ti:Sa) which typically generate pulses with τ = 100–150 fs at τ = 80–100 MHz. They are tunable from 680 to 1, 080 nm.

**Image Formation**

The laser is focused onto the sample and scanned through the specimen in a raster fashion by a scanner, most commonly a pair of galvanometer mirrors (one for each image axis). The image is reconstructed from digitizing the point measurements of the fluorescence intensity at each position in the sample. Emission collection can be efficient and simple because all photons generated are signal and there is virtually no background: even multiply-scattered fluorescence photons can be assigned to their origin due to the localized nature of signal generation. To increase efficiency of fluorescence collection, non-descanned detectors are employed, most commonly large area photomultiplier tubes (PMTs) positioned close to the back aperture of the microscope objective. PMTs allow for the detection of multiply scattered fluorescence photons that may leave the objective lens at randomly divergent angles and are also favored for their high gain and low readout noise.

**Advantages**

A key advantage of TPM over confocal microscopy is reduced attenuation in biological specimens due to reduced scattering and absorption of near infrared light when compared to UV and visible light. Even though Rayleigh scattering is just an approximation in biological imaging, the inverse relationship (~λ^−4^) between scattering and excitation wavelength remains valid. This results in deeper penetration of the pulsed laser source into scattering samples compared to conventional microscopy. Also, the “optical window,” placed at 700–1.000 nm where tissue absorbance is orders of magnitude less than the absorption in the UV or visible region, falls conveniently into the spectral excitation window of TPM. Lower photon absorption in the tissue additionally means lower levels of phototoxicity and sample heating. However it should be noted that within the focal volume, the possibility of bleaching, damage and reduced viability remains.

**Increasing Excitation Efficiency**

Since the fluorescence signal in TPM scales with 1/(τ*R), an increase in signal can be achieved with a reduction in the duty cycle τ*R. A reduction of the repetition rate *R* increases pulse energy, however, a limit is given by the repetition rate since at least one laser pulse must be delivered per image pixel. The reduction of pulse duration τ also leads to increased signal but accurate dispersion correction in the optical train becomes essential. As pulsed laser light traverses the beam path of a microscope, the optical elements slow the shorter wavelength components of the pulse relative to the longer wavelengths, consequently broadening the pulse; a phenomenon known as (positive) group velocity dispersion (GVD). Positive GVD compensation is achieved by introducing optical elements which exhibit negative GVD, introducing an equal but opposite dispersion on the pulse prior to entering the microscope, thus maintaining the shortest pulse duration at the sample plane. Dispersion compensation is often achieved using prisms (Akturk et al., 2006) or chirped mirrors where multiple layers of dielectric material with increasing layer spacing vary the distance traveled by different wavelengths of the pulse and compensate for GVD (Szipöcs et al., 1994). Importantly, GVD worsens the shorter the pulse since the spectral bandwidth of the pulse increases with decreasing pulse duration. A limit to the benefits of shorter pulses is reached when the spectral width of the laser excitation approaches or exceeds the width of the excitation spectrum of the dye ~ 30–40 nm (full width at half maximum).

**The Fundamental Depth Limit**

The imaging depth in TPM cannot be increased indefinitely by increasing the excitation efficiency of the laser. A fundamentally constrained penetration depth restricts investigations in cardiac tissue to a few hundreds of micrometers in mouse left ventricle tissue (Salerno et al., 2019). In turbid media, the ballistic (unscattered) fraction of photons reaching the focal spot decays exponentially with depth. The total power of light reaching a certain depth decays more slowly, approximately as 1/depth. Due to these different decay rates, at depths depending on the scattering mean free path length and anisotropy of the tissue, the peak intensity of ballistic light will fall below that of scattered light, effectively rendering imaging impossible. Even before this depth limit is reached, background fluorescence generated partially by ballistic and mostly by scattered light near the surface will dominate the signal (Theer and Denk, 2006). The fundamental imaging depth in TPM is reached when this out-of-focus (background) fluorescence equals the fluorescence signal emanating from the focal spot; the signal-to-background ratio reaches unity. This fundamental limit to achievable tissue penetration depth is a direct consequence of light scattering by tissue and irrespective of available laser power or potential photodamage.

The ubiquity of optical mapping in cardiac electrophysiological research owes its popularity not only to the high quality and throughput of the data generated, but also the relative simplicity of the setup, particularly with regards to excitation light sources. In contrast, TPM has a relatively high cost and perceived complexity barrier which has restricted its widespread adoption in cardiac electrophysiology. Full commercial microscope systems remain expensive platforms, often bundled with tuneable Ti-sapphire lasers which do not deliver sufficient power to the sample plane for deep tissue imaging at wavelengths within the second IR imaging window (>1000*nm*). Data throughput, particularly for whole heart imaging is also relatively low. Owing to the need for tight spatial focusing, it is technically challenging to combine the high spatial resolution of TPM with larger fields of view and/ or fast volumetric imaging rates. Axial focusing remains slow and limited to the speed of a resonant objective piezo scanner, though remote refocusing (Botcherby et al., 2008; Alfred Millett-Sikking and York, 2018) increases axial scan speed and has been used for fast measurements of sarcomere length and cell orientation in Langendorff-perfused hearts (Botcherby et al., 2013). The following sections discuss these aspects in more detail.

#### 2.5.1. Increasing Imaging Depth

As highlighted in Box 2, a fundamental imaging depth limit exists for a given system, independent of the excitation efficiency of the laser. This is due primarily to tissue scattering effects and increasing background fluorescence generated near the tissue surface (assuming tissue staining is homogeneous throughout the sample thickness). Several optical techniques have been proposed to overcome the TPM depth limit, while retaining scanning rates necessary for optical electrophysiology measurements. Temporal focusing, primarily used to allow video-rate optical sectioning, images a scan line across a blazed diffraction grating to spatially separate the spectral components of the laser pulse, effectively broadening the pulse width. The grating is imaged onto the sample plane via a high-NA objective lens and scanned in one axis to form an image. In theory this should reduce surface background fluorescence because the broadened pulse entering the sample only becomes short enough to permit non-linear interactions at the objective focal plane (Tal et al., 2005). While this approach is successful for rapid-scanning, improved depth imaging is questionable. This is because while point-scanning systems can use all scattered photons to detect an image, only ballistic or weakly scattered photons are useful for image formation with temporal focusing. A direct comparison of temporal focusing microscopy and TPM estimated the latter has up to 2X the penetration depth in highly scattering biological material (Rowlands et al., 2015). Alternatively, differential aberration correction was designed specifically to reduce background fluorescence contamination by placing a deformable mirror into the beam path of a raster scanning TPM which periodically (every second line or frame) introduces an optical aberration pattern to defocus the laser pulse. This preferentially reduces focal excitation while preserving the background. Subtracting the defocused image from the original produces an enhanced contrast image (Leray and Mertz, 2006; Leray et al., 2008). Contrast at a given depth is improved by up to 6X, a potentially significant gain in TPM depth imaging. It should be noted however, that because two images must be formed, the frame rate is effectively halved. A variant of this approach using a delay line and a fixed aberration optic allows near-simultaneous acquisition of two images in separate channels (Xiao and Mertz, 2019). Differential aberration shows promise as a powerful, lightweight approach for deeper TPM imaging and a thorough quantitative evaluation of its usefulness using cardiac muscle is warranted.

#### 2.5.2. The Red Shift

A different approach to increasing the penetration depth of non-linear microscopy techniques is to decrease the effects of scattering itself. Novel engineered fluorophores, whose absorption and emission spectra are shifted toward the IR, take advantage of the decreased scattering of longer wavelengths. However, to image deeper into highly scattering samples, 3PM, the simultaneous absorption of three photons (of 1,300 or 1.700 nm wavelength) to excite fluorescence, has emerged as a promising technique which outperforms two-photon excitation of a different fluorophore at the same wavelength. Due to the higher non-linearity of the 3-photon absorption process, its cross section is several orders of magnitude smaller compared to TPM and this leads to virtually background-free deep imaging and highly improved signal-to-background ratio. However, the extremely low 3PA cross-sections (for example resulting in absorption rates ~20 orders of magnitude lower than 2PA), necessitate higher pulse energies, usually obtained by low repetition rate (2–4 MHz) lasers. The higher peak energy results in higher peak power and therefore an increased risk of non-linear tissue damage whereas the longer wavelength leads to higher absorption and therefore more sample heating.

## 3. Correlation of Structure and Function

A major current technical challenge in optical cardiac imaging is the deterministic correlation between structural features and functional performance at different hierarchical levels, from subcellular components such as individual Ryanodine receptors (RyR) using DNA-PAINT techniques (Hurley et al., 2020) to single isolated CM and intact heart tissue. Assessing the structure and the corresponding local function in a correlative manner is particularly relevant in pathological settings where structural and ultra-structural alterations occur e.g., alteration of excitation-contraction coupling machinery, myofilament disarray or extracellular fibrosis. In the subsequent two sections, two case studies are described to demonstrate the power of optical techniques to measure and assess the function of sub-resolution membrane structures within cardiac muscle cells.

### 3.1. Super-Resolution Techniques Applied to Cardiac Muscle Electrophysiology

The function, morphology, and proteome of atrial (ACM) vs. ventricular (VCM) CM differ significantly (Brandenburg et al., 2016a). Compared to VCM, the ACM's triangle-shaped action potential and phase-2 plateau are markedly shorter, yet followed by a more gradual phase-3 repolarization and a less negative resting membrane potential (Fatkin et al., 2007). In contrast, intracellular Ca^2+^ release in ACM is highly heterogeneous and is frequently described as a slow inward-propagating U-shaped Ca^2+^ gradient (Thul et al., 2012; Greiser et al., 2014). However, contractile activation of atrial compared to ventricular muscle strips occurs significantly faster (Lüss et al., 1999). This results in a conceptual paradox: how is atrial excitation and contraction rapidly coupled, if cell-wide Ca^2+^ release is 1–2 orders of magnitude slower in ACM compared to VCM (Thul et al., 2012; Greiser et al., 2014)? The case study described below demonstrates the use of super-resolution techniques to investigate the details of sub-micron structures in cardiac cells.

**Figure 2:**
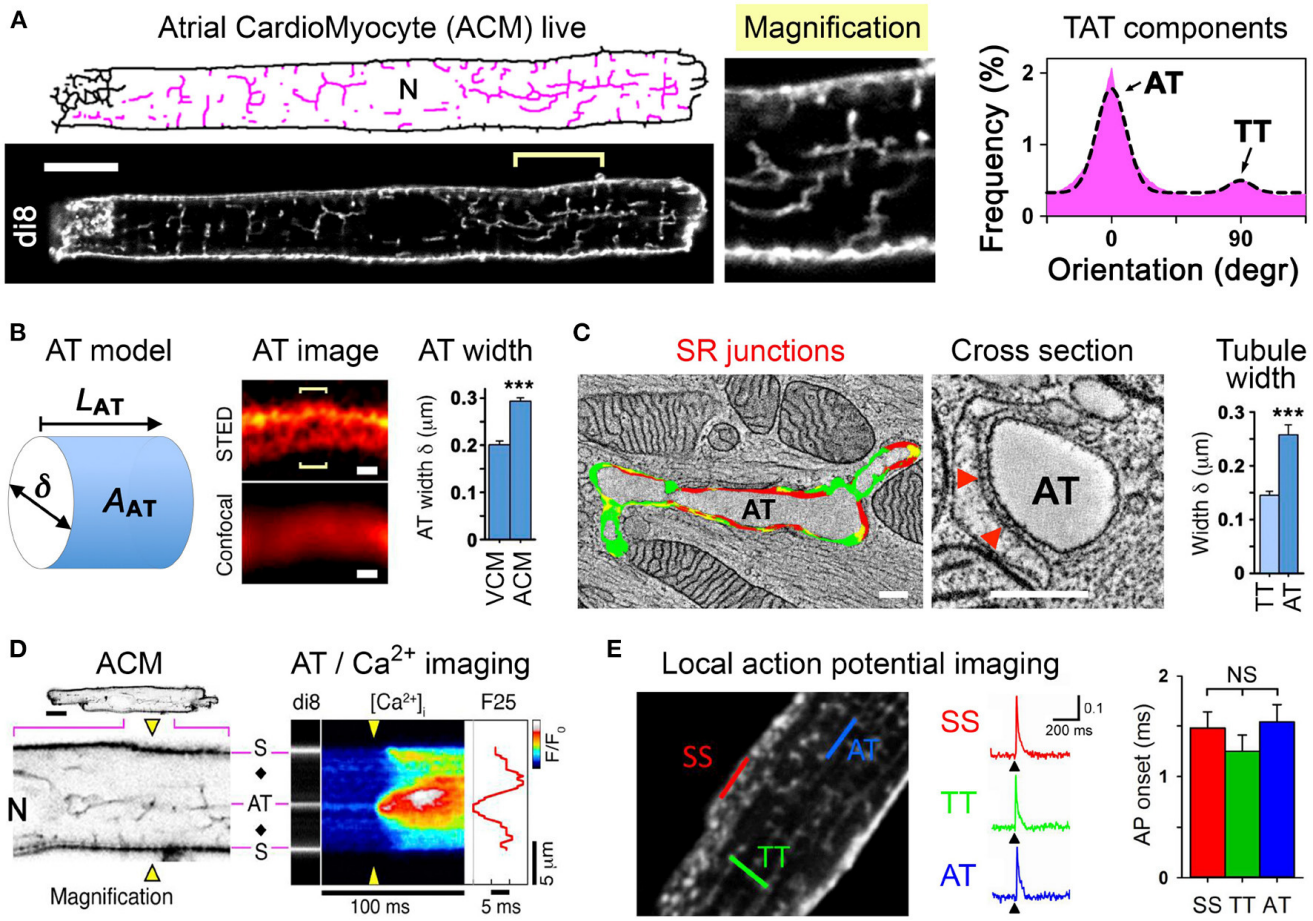
A-tubules provide Ca^2+^ signaling super-hubs for atrial excitation-contraction coupling. **(A)** Confocal live imaging of di-8-ANEPPS stained (di8) TAT structures visualized as skeleton (pink). N, nucleus. Scale bar 10 μm. Histogram showing the TAT component orientations and Gaussian fit. Abundant AT (0°) vs. sparse TT (90°) components. **(B)** Cartoon of a cylindrical AT model. AT width (δ) measurements were used to estimate the surface area (AAT); LAT, AT component length. AT width was determined from optical cross sections (brackets) using the local STED signal distribution and compared between VCM vs. ACM as bar graph. Scales 200 nm. **(C)** Electron tomography images and segmentation of a longitudinally sectioned and a cross-sectioned AT structure. Scales 200 nm. Red color indicating AT-SR junctions ≤ 15 nm in gap width containing RyR2 densities. Red triangles mark two exemplary electron densities compatible with RyR2 channels. **(D)** Confocal negative contrast visualization of intracellular AT structures and transversal line scanning (yellow triangles) of intracellular Ca^2+^ imaging (fluo-4). A field potential-evoked Ca^2+^ transient is activated at AT and subsurface (S) structures; black diamonds, off-membrane cytosolic sites; F25, Ca^2+^ signal onset at 25 % signal amplitude; $\Delta F/F_{0}$, normalized fluorescence intensity ratio as indicated by look-up-table; N, nucleus. **(E)** Two-photon action potential recordings from specific TAT components labeled with the voltage-sensitive dye di-4-ANE(F)PTEA (2 μM). Normalized fluorescence traces ($\Delta F/F_{0}$) were recorded from the scan regions indicated by color. At AT, TT, and SS membranes structures the simultaneous action potential activation upon pacing (black arrowheads) is apparent. Grouped bar graph showing no significant difference for action potential onset (the time interval between the end of the stimulus and the rise of the fluorescence signal above a threshold of 4 % $\Delta F/F_{0}$. AT, A-tubule; TT, T-tubule; SS, subsurface membrane site. NS, not significant. Adapted from Brandenburg et al. (2016b).

In rodent ACM typically no or only few residual T-tubules are reported (Kirk et al., 2003). In contrast, VCM are characterized by a high density of T-tubules, where each may rapidly propagate electrical signals (see also section 2.4). To combine cell isolation with high-resolution imaging workflows for ACM, fluorescent dyes that selectively intercalate in the extracellular membrane leaflet are highly attractive to assess the local membrane structure and integrity (Wagner et al., 2014; Brandenburg et al., 2018). Surprisingly, when implementing stringent cell quality and confocal imaging workflows, 100% of ACM isolated from mouse hearts were shown to exhibit a unique cell-wide transverse-axial-tubule (TAT) membrane network (Figure 2A, left) (Brandenburg et al., 2016b). Importantly, analysis of the TAT components revealed that axially oriented A-tubules dominate the ACM membrane network, whereas T-tubules are far less abundant (Figure 2A, right).

Interestingly, confocal imaging failed to locally resolve di-8-ANEPPS stained A-tubule cross-sections, whereas STimulated Emission Depletion (STED) superresolution nanoscopy improved local contrast sufficiently to determine the width (δ) of intact A-tubules in living ACM (Figure 2B). On average, the A-tubule width was significantly larger in ACM compared to VCM (Figure 2B) (Brandenburg et al., 2016b), suggesting that A-tubules exhibit a much larger cytosolic membrane surface area (AAT). Indeed, electron tomography of high-pressure frozen ACM showed that A-tubules exist as large cylindrical membrane structures that form extensive SR junctions densely filled with RyR2 Ca^2+^ release channels (Figure 2C, left) (Brandenburg et al., 2016b). Finally, quantitative analysis confirmed that A-tubules exhibit approximately 100 nm larger diameters than T-tubules in ACM (Figure 2C, right).

Using immunostaining of ACM, voltage-dependent L-type Ca^2+^ channels were identified in A-tubules: Cav1.2 clusters were shown to occur at a higher density in A-tubules compared to T-tubules (Brandenburg et al., 2016b), and additionally Cav1.3 clusters were localized in A-tubules (Brandenburg et al., 2018). Consistent with abundant L-type Ca^2+^ channel clusters in A-tubules, intracellular Ca^2+^ imaging combined with di-8-ANEPPS staining showed field pacing-evoked rapid Ca^2+^ release gradients around A-tubules, whereas surface membrane and particularly cytosolic sites each showed a significantly increased Ca^2+^ release latency (Figure 2D) (Brandenburg et al., 2016b).

Finally, RAMP was used for local *in situ* action potential recording at specific TAT sites in living ACM (Figure 2E, left and section 2.4). Strikingly, action potentials recorded locally at A-tubules, T-tubules, or subsurface sites showed the same rapid onset (Figure 2E, right), amplitude and upstroke velocity (Brandenburg et al., 2016b). Thus, despite a dramatically lower T-tubule density (Figure 2A, right) and a relatively large distance from the cell surface, voluminous A-tubules appear to function very effectively as electrical conduits for rapid action potential spread deep inside ACM.

While the physiological and clinical relevance of these new atria-specific A-tubule findings is still emerging, several important implications are obvious: (1) ACM express a cell-specific electrically excitable TAT network quite different from VCM; (2) the discovery of A-tubules as dominant excitable membrane conduits provides a new model of “super-hub” Ca^2+^ signaling and ACM-specific excitation-contraction coupling; (3) in addition to Cav1.3 channels thousands of proteins are differentially expressed in ACM (Brandenburg et al., 2016a), yet none of the clinically relevant ion channels and G-Protein coupled receptors have been mechanistically established in the context of A-tubule membrane organelles so far.

As functional L-type Ca^2+^ channel changes represent a disease mechanism of atrial fibrillation, major future questions are how Cav1.2 and Cav1.3 clusters are organized locally and relatively to RyR2 channel Ca^2+^ release sites, and which specific voltage-dependent roles the atrial L-type Ca^2+^ channel isoforms exert in A-tubules. Interestingly, while the Cav1.2 interacting protein junctophilin-2 is expressed five-fold less in the atria compared to the ventricles, it is highly abundant inside sarcoplasmic reticulum (SR) junctions along A-tubules (Brandenburg et al., 2019). Thus, non-invasive superresolution optical methods are not only essential for establishing differential Cav1.2 and Cav1.3 functions in ACM, but they will be instrumental for identifying early disease-causative mechanisms of atrial cardiomyopathy preceding atrial fibrillation and stroke (Goette et al., 2016).

### 3.2. Fluorescence Recovery After Photobleaching to Probe Structure and Function of the T-Tubular System

The optical technique of fluorescence recovery after photo-bleaching (FRAP) microscopy is a useful method for examining the diffusion of probes within limited structures within the cell (Bers and Shannon, 2013). In relation to examining the diffusion within the TAT structure, FRAP has been used to demonstrate marked species differences in the tortuously and therefore lumenal diffusion of solutes within these membrane structures (Kong et al., 2018). In this study the authors used FRAP microscopy to probe the diffusion properties of TAT: fluorescent dextran within the TAT system lumen was photo bleached and signal recovery by diffusion of unbleached dextran from the extracellular space was monitored. Remarkable is how an optical-based imaging approach can support or implement structural data hitherto achievable only with 3D electron tomography (Rog-Zielinska et al., 2018). More in general, combining FRAP with detailed computational modeling the authors designed a new pathway to understanding how TAT remodeling may contribute to altered cellular function. In this respect, the combination of FRAP with optical voltage measurements from TAT membranes has also offered the opportunity to study the relationship between structural alterations of the T-tubules and defects of action potential propagation found in the failing heart.

For instance, in a recent study, Scardigli et al. (2017) exploited the formal analogy between diffusion and electrical conductivity to link the latter with the diffusion properties of the TAT. The time constant of fluorescence recovery was correlated with the apparent diffusion coefficient of the fluorescent molecules and linked to electrical conductivity. These data were used to evaluate the efficiency of the passive spread of membrane depolarization along TAT system. The characteristic length constant of T-tubules was calculated as approximately 300 μm: an order of magnitude larger than the cellular radius, indicating a remarkable safety factor of the normal TAT system structure in passively conveying membrane potential changes across the cell.

Further refinement of the FRAP technique was then employed to address the role of the mechanical activity associated with contraction in the cardiac cell with the enhanced movement of solutes including ions through the tortuous TAT system driven by advection-assisted diffusion (Rog-Zielinska et al., 2021). This phenomena is thought to offset the accumulation of solutes within the TAT system as a result of changes in heart rate (Rog-Zielinska et al., 2021). This novel finding provides another potential mechanism for dysfunction, namely the reduced capacity for solute exchange that may result from a remodeled TAT system in failing hearts.

### 3.3. Light-Sheet Microscopy for Mesoscale Structural Imaging

Taking correlative work to another order of magnitude, extremely challenging current experiments aim to dissect morpho-functional relationships between cellular organization and functional abnormalities at the whole organ level. At the tissue level, current studies generally associate structural alterations with the corresponding electromechanical dysfunction without showing real cause-effect relations. What is missing is a direct link between the functional study and quantification of tissue disorganization performed on the same tissue. The main limitation is the inefficacy of standard imaging methods and staining approaches in performing high resolution imaging (cellular or sub-cellular resolution) in tissue. During the past years, different methods have been developed for clearing fixed tissue. Most of them, however, present several limitations such as tissue shrinkage, structural alteration, fluorescence quenching and incompatibility with immunostaining (Silvestri et al., 2016). The challenge of producing large, transparent and fluorescently-labeled volumes has been overcome recently by applying a tissue transformation approach, CLARITY, and it's related derivatives (Chung et al., 2013; Pianca et al., 2019; Olianti et al., 2020, 2021). These methods allow high transparency, immunolabeling and structural and molecular preservation. One drawback with the current techniques is the poor penetration rates of the tissue by antibodies, to the extent that many months of incubation are sometimes required depending on tissue dimensions.

Clearing methods render biological tissue transparent by equilibrating the refractive index throughout a sample to reduce inhomogeneities in light scatterers (Richardson and Lichtman, 2015). Different approaches have been proposed based on organic solvents, high-refractive index aqueous solutions, hyperhydrating solutions and tissue transformation (Silvestri et al., 2016). Most notable amongst the latter approaches, CLARITY (Chung et al., 2013) employs hydrogel embedding of the sample by perfusing tissue with a fixative, a temperature-sensitive crosslinker and the hydrogel monomer which forms covalent links with native proteins. After fixation, the tissue of interest is warmed to induce cross-linking of the monomers into a nanoporous hydrogel mesh. Lipids are then removed from the sample with a detergent solution either passively, or actively through electrophoresis. The lipid-free samples are then incubated in refractive index matching solutions for clearing. This method in combination with advanced imaging techniques such as light-sheet or two-photon microscopy (see Box 2) allows 3D reconstruction of substantial heart tissues at high resolution.

Light-sheet microscopy, originally described in 1903 (Siedentopf and Zsigmondy, 1903) and re-invigorated in the early 2000s (Huisken et al., 2004), achieves optical sectioning over the entire field of view by selectively illuminating a thin slice of tissue with a sheet of light which is positioned to coincide with the focal plane of a perpendicularly placed microscope objective for fluorescence detection. In contrast to point-scanning epi-illumination methods like confocal or TPM, this uncoupled illumination—detection scheme has two major advantages. Firstly, the detection scheme is essentially that of a wide-field microscope and hence affords much faster acquisition rates through the registration of multiple points in parallel rather than sequentially. Secondly, the selective illumination greatly reduces unnecessary photoactivation and bleaching of fluorophores above and below the focal plane. However, this very same uncoupling of excitation and detection makes light-sheet microscopy particularly sensitive to tissue scattering and the technique thus requires samples that are either intrinsically transparent (e.g., Zebrafish larvae) or else an opaque tissue that has been made transparent through tissue clearing. If tissue is intrinsically transparent, it is possible to combine structural observations in live tissue, even over extended periods of time (for example to track every single cardiac cell during early ontogenetic development in the Zebrafish heart) with functional Ca^2+^ imaging (Weber et al., 2017; Taylor et al., 2019).

This mesoscopic imaging may help to elucidate the role of cellular organization in cardiac electro-mechanical (dys)function and pave the way for a unifying approach which integrates functional and structural data. Such an experimental model would enable a comprehensive investigation of the morphological changes that lead to electrical and mechanical alterations after disease-associated structural remodeling.

## 4. The Use of Light to Manipulate and Measure Cardiac Electrophysiology

Extending the application of optical techniques in cardiac applications, optogenetics has added the capability to manipulate cardiac function by light (see review Entcheva and Kay, 2021). Optogenetics combines targeted light stimulation and the genetic expression of light-sensitive ion channels or pumps to enable cell-specific, non-contact stimulation with sub-millisecond temporal and micrometer spatial resolution. The characterization of the non-selective cation channel Channelrhodopsin2 (ChR2) in the early 2000s (Nagel et al., 2002) initiated the explosive growth of field of optogenetics. Compared to electrical stimulation, in excitable cells expressing ChR2, pulsed illumination induces inward currents which not only trigger excitation but can also precisely modify the shape of action potentials (Park et al., 2014; Williams and Entcheva, 2015). Light responsive Cl^−^ and H^+^ pumps, as well as K^+^ and Cl^−^ ion channels allow light-induced hyperpolarization of cardiac tissue. Direct optogenetic hyperpolarization with patterned light has been used to provide mechanistic information about circuits involved in cardiac pacemaking (Arrenberg et al., 2010), or to affect cardiac arrhythmias by selective expression in sympathetic neurons in a canine model of post-ischemic electrophysiological remodeling (Yu et al., 2017). The ability to bi-directionally manipulate cardiac function (to excite and to inhibit) using light, and the possibility to combine these newer optical tools with optical mapping have afforded new means to address mechanistic and translational questions related to cardiac arrhythmias (Entcheva and Bub, 2016).

### 4.1. Optogenetic Cardioversion/Defibrillation: Progress and Challenges

Beyond optogenetic pacing demonstrated in early cardiac studies (Arrenberg et al., 2010; Bruegmann et al., 2010; Jia et al., 2011), proof-of-concept studies for optogenetic cardioversion/defibrillation have been shown in transgenic mice and wild type mice and rats after AAV-mediated expression of ChR2 (Bruegmann et al., 2016, 2018; Crocini et al., 2016a; Nyns et al., 2016). The light parameters (light intensity, pulse duration, etc.) required for successful termination have been determined using many different approaches for illumination (optical fiber/global LED, DMD, and μLED) and computational modeling. Using this range of techniques, different excitation patterns are possible, opening the way for the investigation of a range of defibrillation approaches including: (i) targeted pacing to fill the excitable gap or (ii) continuous illumination for prolonged phases of depolarization with the aim of a complete conduction block. Interestingly, conduction block seemed to be more effective but required higher light intensities compared to targeted pacing when illumination was performed randomly without analysis of the underlying arrhythmia pattern (Bruegmann et al., 2018). However, knowing the underlying reentry wavefronts including the spatial extent of excitation gaps or global illumination of the whole ventricular surface might enable a reduction in the light intensity requirements (Crocini et al., 2016a). For targeted pacing, feedback loops between sensing electrodes or optical mapping and light stimulation have been developed which permit testing numerical hypotheses for more effective, gentle cardioversion/ defibrillation (Hussaini et al., 2021). The translation to larger animals including humans has been investigated using, *in silico* modeling. This work indicates that in principle, the optogenetic route is feasible and suggests defibrillation mechanisms distinct from that achieved by conventional chest paddles (Bruegmann et al., 2016; Hussaini et al., 2021). However, there are considerable technical issues to overcome including: (i) the need for gene transfer, (ii) the expression of non-mammalian opsins in humans and (iii) implantation of light sources. Nevertheless, the gain would be pain-free, tissue-sparing and efficient arrhythmia termination in the clinic.

### 4.2. Real-Time Manipulation of Cardiac Electrical Activity in a Closed-Loop System

**Figure 3:**
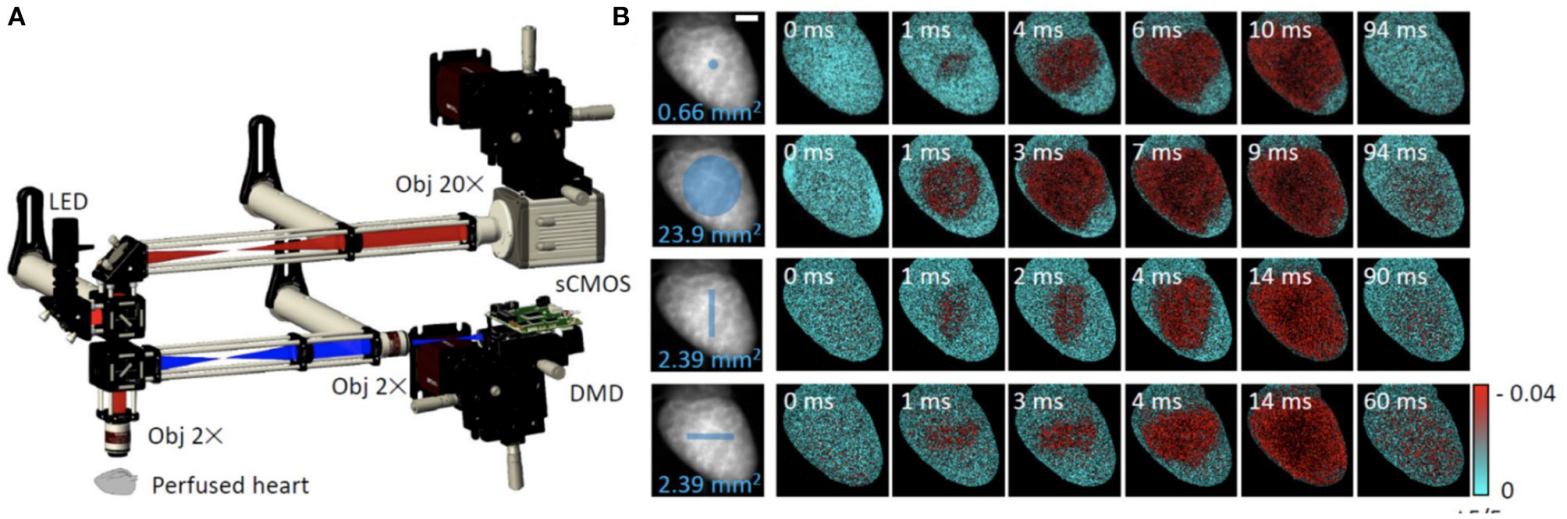
**(A)** Optical setup for real-time closed-loop optogenetic stimulation with a digital micromirror device (DMD) in response to ongoing cardiac activity, simultaneously optically mapped with a sCMOS camera. **(B)** Exemplary customizable stimulation pattern and cardiac excitation wavefronts. Modified from Scardigli et al. (2018b).

All-optical cardiac electrophysiology is a powerful contact-less interrogation and manipulation technique (Entcheva and Bub, 2016; Scardigli et al., 2018b). A recent example of the capability of optical voltage reporters combined with optogenetic actuators at the whole heart level is the development of a wide-field mesoscope to simultaneously optically map and manipulate action potential propagation in whole mouse hearts (Scardigli et al., 2018b) (Figure 3A). A computer-controlled digital micromirror device (DMD, see Box 3) capable of drawing arbitrarily-chosen illumination patterns was used to create precise spatial and temporal patterns of optogenetic stimulation on the epicardial surface of isolated hearts (Figure 3B). Operating in closed-loop with a feedback-control strategy, this system can react in real-time to ongoing electrophysiology as revealed by optical mapping, opening the prospect for real-time resynchronization therapy and cardiac defibrillation. These techniques can serve as a “biological stimulator” (for example by optical stimulation of ChR2), or as a “biological inhibitor” (when using optically-activated K^+^ flux systems) to inform new *in vivo* strategies for controlling arrhythmias. Such a system is an ideal test-bed to investigate strategies to defibrillate or interrupt tachy-arrhythmias using the minimal power and area of illumination with a view to advancing our knowledge of the electrophysiology of arrhythmias that may be translatable to the clinics.

Box 3 **Digital Micromirror Devices**

A digital micromirror device, or DMD, is a micro-opto-electromechanical system comprised of millions of microscopically small moving mirrors to create a video display of the type now found in digital projectors. The mirrors on the DMD are arranged on a μm diamond pixel geometry and by applying a tilt angle of ±12°, each micro-mirror can be switched on or off individually. A high numerical aperture relay system can be used to project the DMD pattern onto the sample. DMDs have been employed for controlling cardiac activity at fine spatial and temporal scales using light in cultured cardiac monolayers and whole-heart preparations. These light projectors offer sub-millisecond temporal resolution and have been used to demonstrate fine control of tissue functional properties, including wave velocity (Burton et al., 2015), or to embed high temporal resolution “small” images into larger but more slowly acquired still image sequences (temporal pixel multiplexing, Bub et al., 2010).

### 4.3. Wave Control to Improve Arrhythmia Management and Termination

Present-day electronic cardiac devices include cardiac pacemakers (to maintain normal rhythm) and cardioverter-defibrillators to terminate arrhythmias and restore normal rhythm. With newer optical technologies, including optogenetic modulation of cardiac function, conceptually new strategies for control have been proposed that are not possible with classic electrical means. Using optogenetic actuation and dye-free optical mapping, it was demonstrated that the direction, speed and chirality of cardiac excitation waves can be modulated by light (Burton et al., 2015). To achieve this, a quasi-real time feedback was applied with dynamically-modulated light patterns using a DMD. Space-time varying illumination enables “wave steering” (Entcheva and Bub, 2016) and can bring insights for finer arrhythmia control in the clinic with lower-energy approaches and devices. Until recently, the only options for wave control have been pharmacological (adding exogenous compounds that alter cardiac channel function) or electrical (using an electrode or an array of electrodes to stimulate the tissue). These approaches have limitations as they either change wave properties in the entire tissue or at a limited number of locations due to physical limits on the density and number of electrodes in an electrode array. All-optical electrophysiology offers the ability to deliver dynamic space-time patterns of light for stimulation/suppression, and the possibility for real-time feedback control of macroscopic emergent properties, i.e., cardiac waves of excitation. Wave control goes beyond the simple initiation and termination of excitation waves (pacing and defibrillation/cardioversion) and the associated frequency modulation. Instead, using all-optical means, one can focus on manipulation of the macroscopic (emergent) properties. Recent examples include termination of arrhythmias through re-entrant wave drift induced by longer low-light pulses (Hussaini et al., 2021). While high spatial and temporal resolution optogenetic manipulation in the intact heart *in vivo* is still not feasible, optogenetic tools yield, for the first time, an experimental platform to rapidly test anti-arrhythmic strategies in *ex-vivo* preparations. This offers the flexibility to manipulate the electrophysiological variables within the heart hitherto only possible in computational models.

### 4.4. Cell Specific Optogenetics to Interrogate the Myocardial Network

**Figure 4:**
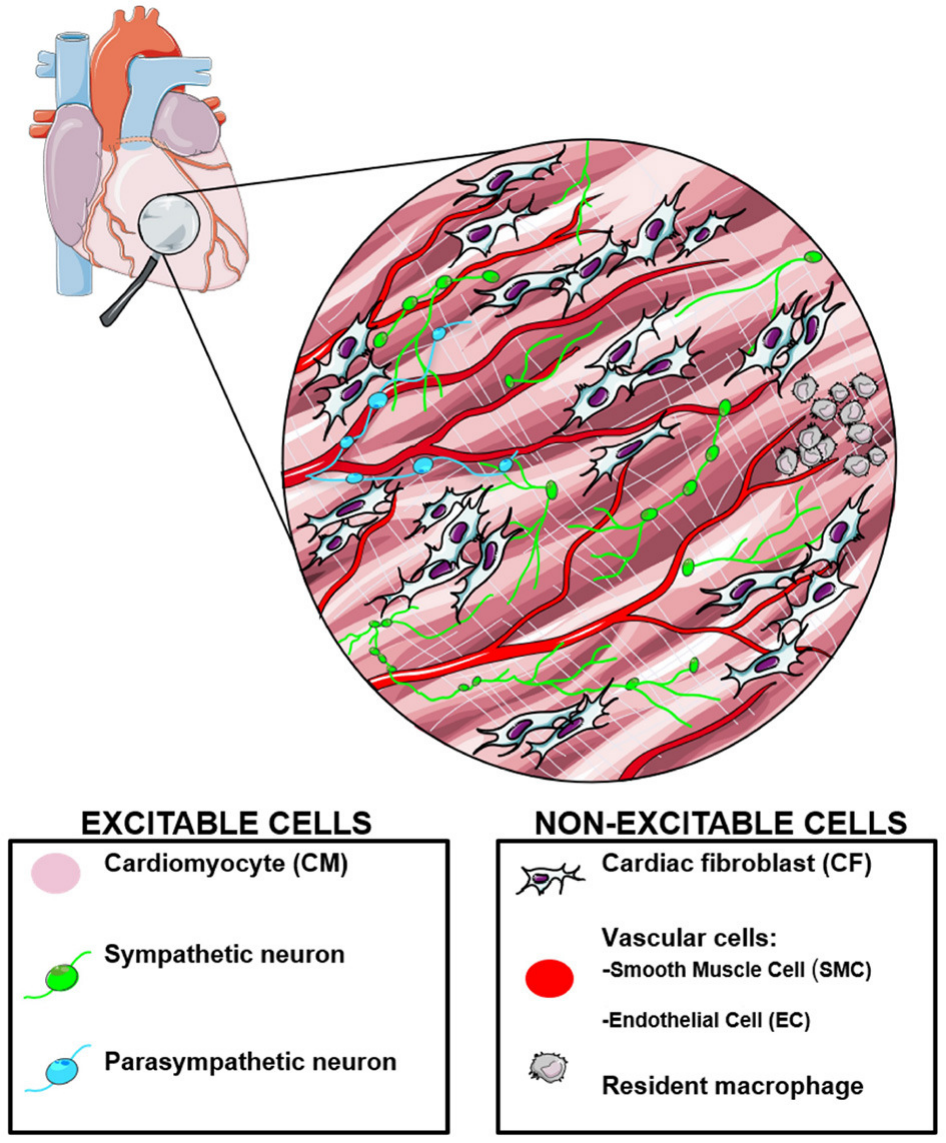
The heart is a multicellular network of excitable and non-excitable cells, which have been targeted with various opsins for optogenetic investigation. Modified from Zaglia et al. (2019).

Different from conventional electrophysiological approaches, optogenetics, by its nature, is cell type specific, as sensitivity to photostimulation or optical reporting of function are achieved by transgene expression. This feature was immediately exploited in neuroscience, as it enabled the functional dissection of neuronal circuits in the multiplexed network forming the central nervous system. An emerging concept of the heart as an interconnected multicellular system is a prerequisite to formulate the appropriate physiological questions optogenetics can answer (Figure 4). Heart function is based on the anatomical and functional interaction between excitable (i.e., working and conducting CM), non-excitable conductive [e.g., cardiac fibroblasts (Quinn et al., 2016) and macrophages (Hulsmans et al., 2017)], and extrinsic excitable (i.e., cardiac autonomic neurons) cells, and non-excitable modulators (i.e., endothelial and smooth muscle cells) (Zaglia et al., 2019). Cell-specific optogenetic stimulation within intact heart has first been demonstrated in a proof-of-principle study by Bruegmann and colleagues (Bruegmann et al., 2010), and subsequently this approach was used to infer the contribution of working and conducting CM to arrhythmias in the normal and ischemic myocardium. While alterations in the Purkinje Fiber (PF) network, including both PF cell dysfunction and PF coupling to the working CM have been suggested in several acquired or genetic cardiovascular diseases, assessment of PF function is only indirect and non-specific with conventional electrophysiological techniques. Cardiac optogenetics has allowed direct investigation of the source-sink relation in the conduction system vs. working myocardium, and by combining it with a 3D morphological study of the PF network, to determine that the high arrhythmogenicity of the right ventricular outflow tract depends on the peculiar displacement of the terminal PF network (Zaglia et al., 2015).

Another excitable cell population, critical for regulating cardiac function, is represented by autonomic neurons, which densely innervate the myocardium, including extracardiac sympathetic and parasympathetic neurones and a dense network of cardiac ganglia (Julian et al., 2021), influencing potentially all cardiac cell types (Franzoso et al., 2016). It is increasingly evident that neuronal remodeling (i.e., changes in distribution and function of cardiac innervation) takes place upon physiological (e.g., exercise, pregnancy, aging) and pathological (e.g., ischemic damage, heart failure) conditions. *In vivo* assessment of cardiac autonomic neuron function is technically challenging and non-selective. Several groups have used optogenetics to achieve control of cardiac sympathetic (Wengrowski et al., 2015; Yu et al., 2017; Prando et al., 2018; Burton et al., 2020) or parasympathetic (Moreno et al., 2019; Rajendran et al., 2019) neurons, to assess their effects on heart function, to study the biophysics of neuro-cardiac communication and to prevent sympathetic neurons from triggering arrhythmias in post-ischemic electrophysiological remodeling.

Furthermore, in addition to the studies of excitable cells, the method has recently been used in approaches to investigate non-myocyte cell populations. Non-CM cells in the myocardium are mostly inexcitable, and they have classically been relegated to forming the cardiac vascular bed (i.e., endothelial cells, smooth muscle cells) or regulating the extracellular compartment (e.g., fibroblasts). Rapidly accruing evidence has shown that these cell types play additional roles in paracrine and endocrine signaling, and may influence cardiac activity. In addition, some 25 years ago, it had been suggested that cardiac fibroblasts may potentially couple to CM via connexins, affecting cardiac electrophysiology (Kohl et al., 1994). This hypothesis was eventually confirmed in native tissue, using optogenetic expression of GEVI in cardiac non-myocytes (Quinn et al., 2016). Furthermore, expression of Channelrhodpsin-2 (ChR2) or Archaerhodopsin (ArchT) was achieved in primary cardiac fibroblasts, and heterocellular coupling with CM was further investigated (Yu and Entcheva, 2016; Funken et al., 2020; Simone et al., 2020; Boyle et al., 2021; Kostecki et al., 2021). ChR2 expression in smooth muscle cells of the coronary vessels has shown to be a potent tool to control vascular tone, demonstrating local and rapidly induced light-activated vasospasm (Wu et al., 2015). In parallel, selective expression of ChR2 in endothelial cells has shown that modulation of endothelial cell membrane potential can also trigger local vasospasm (Zhang et al., 2015), suggesting heterocellular coupling between the two important cellular components of the vessel wall. Optogenetics was exploited to study the biology of resident heart macrophages, which were shown to electrically communicate via connexins with atrio-ventricular node cells and to influence conduction (Hulsmans et al., 2017). Thus optogenetics revealed novel functions, especially related to heterocellular coupling, of smooth muscle cells, endothelial cells and macrophages.

Long term cardiac studies or experiments in conscious, freely moving animals are technically more demanding than in brain research, due to obvious differences in anatomical constraints. In particular, continuous movement of the heart hampers the stable implantation of light-emitting devices (for example, optrodes which have been developed for deep brain stimulation have limited use in the heart) (sta, 0000). These limitations can be overcome by progress in bioengineering aimed at the development of biocompatible and flexible light emitting tools to be positioned on the epicardial surface or around cardiac neurons (Gutruf et al., 2019). Collaboration with medical physicists would also allow simultaneous optogenetic stimulation of CM or cardiac neurons with the assessment of cardiac function by echocardiography or other imaging methods such as cardiac magnetic resonance. Another limitation, which will presumably be resolved in the next few years, is that, up to now, cardiac optogenetics has mainly been used in rodents, while very little has been performed in bigger animal models (e.g., pigs, sheep), which may be more informative for their closeness to human electrophysiology. In general terms, the clinical or experimental applicability in humans is not immediate, as it requires expression of an exogenous gene in the heart, and implantation of a light emitting source. Rapid progress in molecular biology has yielded viral vectors encoding opsins suited to the use in humans (FDA-approved, ClinicalTrials.gov NCT02556736), and opsin expression in patient hearts may thus be foreseen.

Optogenetic inhibition of autonomic sympathetic neurons is theoretically suited to ablate the adrenergic component favoring or sustaining arrhythmias. The further development of this concept will presumably be utilized to study arrhythmogenesis in genetic or acquired cardiovascular diseases in which adrenergic stress has a key role (catecholaminergic polymorphic ventricular tachycardia, arrhythmogenic cardiomyopathy, Takotsubo cardiomyopathy). While several opsin variants with specific advantages have been used in neurosciences (sta, 0000), cardiac optogenetics has thus far, exploited almost exclusively ChR2 with some exceptions (Kopton et al., 2018; Sierra et al., 2018). It is to be expected that in the next years many newly developed photoactivatable tools will be used in heart studies. In addition to channel-forming opsins, signaling opsins have been developed for spatially and temporally accurate photo-interrogation of signaling pathways in cells/ *in vivo*.

Proof of concept examples have shown light-modulation of *G*_*q*_ and *G*_*s*_ signaling pathways (Beiert et al., 2014; Makowka et al., 2019). Specifically, optogenetic stimulation of the adrenergic *G*_*s*_ signaling cascade using JellyFish Opsin in cultured CM and in the heart of transgenic animals was shown to have a very high light sensitivity, fast kinetics and high spatial resolution (Makowka et al., 2019). The precise time point of activation by triggered illumination allowed to identify differential downstream kinetics of cAMP/PKA effects on pacemaking (positive chronotropy), sarco/endoplasmic reticulum Ca^2+^-ATPase activation (positive lusitropy) and Ca^2+^ induced Ca^2+^ release mechanism (positive inotropy). Furthermore, local illumination allowed to identify regions in the heart which are more sensitive to pathological adrenergic effects such as the left atrium, in which arrhythmogenic premature contractions were induced by localized optogenetic *G*_*s*_ stimulation (Makowka et al., 2019). Importantly, in this special issue of Frontiers in Physiology, Cokic et al. show that long wave opsin enables control of the inhibitory *G*_*i*_ signaling pathway in CM using red light (625 nm), which is spectrally compatible with a blue light sensitive (450–550 nm) optogenetic actuators or reporters (Cokic et al., Optogenetic stimulation of *G*_*i*_ signaling enables instantaneous modulation of CM pacemaking, Cokic et al., this issue). Thus, the combination of Jellyfish Opsin with long wave cone Opsin will allow alternating activation of *G*_*s*_ and *G*_*i*_ signaling to study the effect of dysbalanced sympathetic and parasympathetic vegetative nerve input on the heart which may be involved in cardiac pathologies. Using implantable light emitting devices and optogenetic G protein-coupled receptors it will be possible to study acute and chronic effects of *G*_*s*_, *G*_*i*_ or *G*_*q*_ stimulation on cardiac remodeling and electrophysiology including arrhythmia, hypertrophy or heart failure.

### 4.5. Immediate Translational Application of All-Optical Cardiac Electrophysiology

The pharmaceutical industry and associated regulatory authorities require that all drugs are tested for their cardiotoxic effects before being released for human testing. Early, comprehensive and inexpensive detection of pro-arrhythmic effects based on inhibition of ion channels that control excitability and action potential duration can save the industry considerable costs (Gintant et al., 2019). The CIPA (Comprehensive *In vitro* Pro-arrhythmia Assay) initiative has stimulated the development of newer testing platforms that can provide better predictive value for drug development. The features of an assay required by this sector are: (i) standardized cardiac cells/ tissue and protocols, (ii) scalability and medium-high throughput, i.e., hundreds of assays/ system/ day, and (iii) excellent predictive value relevant to human use. Over the last 10 years commercial sources of human iPSC-CM have become available as a source of standardized human myocardial material. Currently the electrophysiology of monolayer cultures human hiPSC-CM is the most common preparation with electrical activity of the monolayer assessed from an array of monopolar extracellular electrodes (micro-electrode array) embedded into the base of the culture dish. A limitation of these field potentials generated by the intrinsic spontaneous electrical activity of the coupled monolayer of cells is the poor capture of the repolarization dynamics, including action potential duration and shape, which are perceived as some of the most predictive indicators of pro-arrhythmic responses. Alternatively, ion channel responses are tested with planar automated patch clamp systems that offer scalability compared to the traditional gold standard to quantify electrophysiology. Both approaches, used by the pharmaceutical industry, have limitations stemming from the requirement for close contact between the cells and the electrodes for generating meaningful signals, which makes their use with iPSC-CM somewhat problematic.

**Figure 5:**
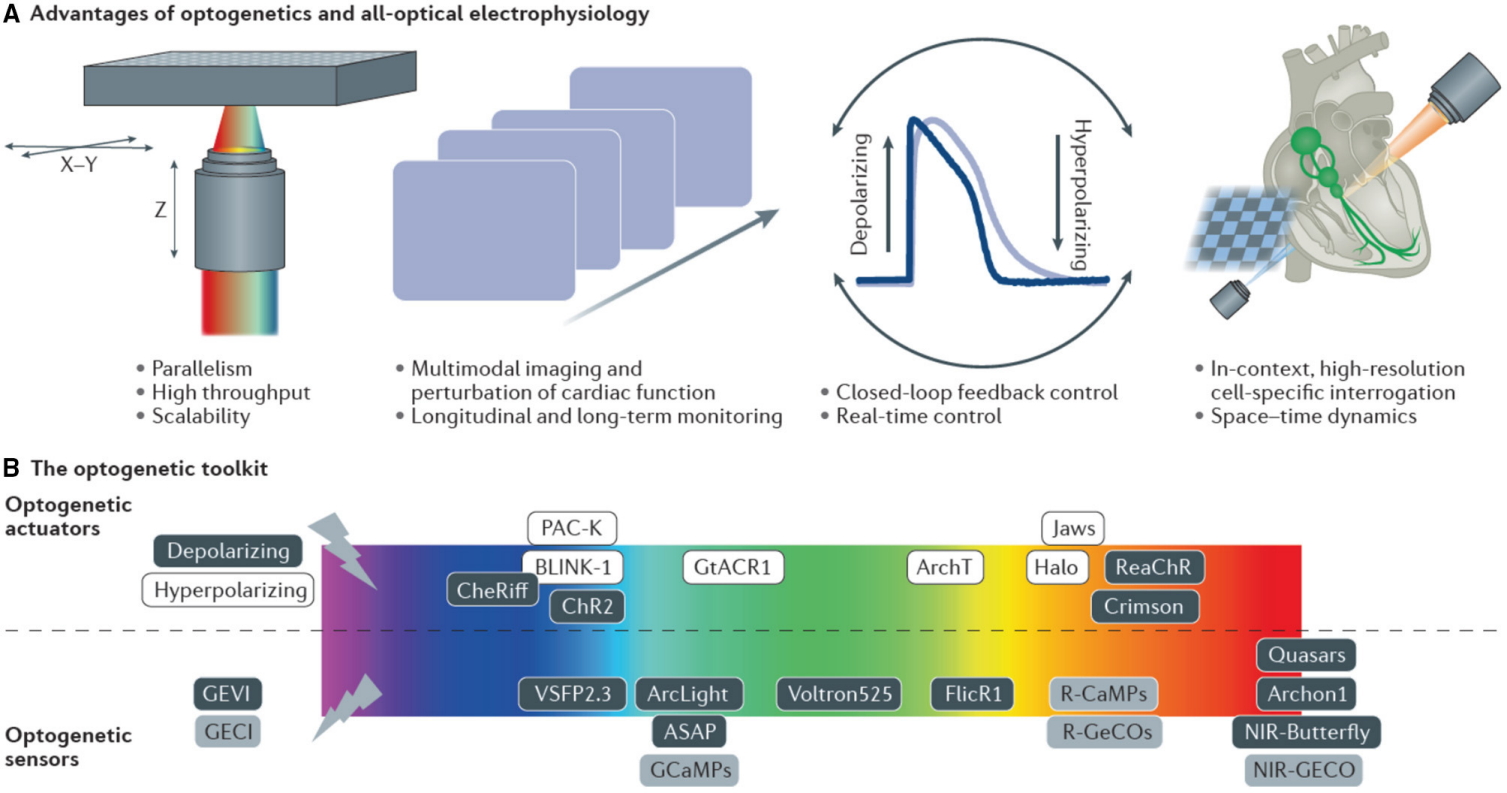
All-optical cardiac electrophysiology and the toolkit of optogenetic actuators and reporters. **(A)** Advantages of all-optical cardiac electrophysiology using optogenetic tools. **(B)** Spectral compatibility of optogenetic actuators and reporters for voltage (GEVI) and calcium (GECI) with the possibility for bi-directional control (depolarization and hyperpolarization) of cardiac excitation. Reproduced with permission from Entcheva and Kay (2021).

Optical technologies for recording and for stimulation offer several advantages to address these challenges. As shown in Figure 5A, these advantages include built-in scalability and high-throughput due to: (i) contactless nature, (ii) high-content functional information through multi-modal imaging and control, (iii) longitudinal observations and (iv) the ability for closed-loop control within multicellular context. Optogenetic pacing has been combined with microelectrode array recordings for better control of the frequency (restitution) responses to drugs of human iPSC-CM (Lapp et al., 2017). Voltage-sensitive and calcium-sensitive dyes have been used in commercial pre-clinical assay platforms to quantify pro-arrhythmic responses to drugs (Hortigon-Vinagre et al., 2016; Pfeiffer-Kaushik et al., 2019).

Optogenetics-inspired developments of automated high-throughput platforms for drug testing using all-optical cardiac electrophysiology have been deployed in conjunction with human iPSC-CM (Dempsey et al., 2016; Klimas et al., 2016, 2020; Werley et al., 2017; Credi et al., 2021). These platforms incorporate spectrally-compatible optogenetic actuators with optogenetic and/or optical reporters for voltage and calcium (Figure 5B) to provide comprehensive characterization of electromechanical responses to drugs. Some of these systems have already found their way into the industrial setting. The immense information content from such high-throughput platforms, combined with machine learning algorithms can yield new hybrid *in vitro - in silico* methods for drug development and for better mechanistic understanding of cardiac arrhythmias. Furthermore, dye-free, completely non-invasive approaches (Ashraf et al., 2021), together with microfluidics-enabled high-throughput platforms (Wei et al., 2020) can allow the chronic longitudinal monitoring of human iPSC-CM. These techniques will facilitate studies on maturation processes, cardiac disease modeling and tissue regeneration.

## 5. Functional Studies in actively Contracting Cells and Whole Hearts

As pointed out before (e.g., section 4.3), imaging of “a moving target” poses significant challenges. In cardiac research, use of mechanical constraints that reduce tissue motion relative to the imaging system, or application of electro-mechanical uncouplers (such as blebbistatin, see section 2.1) have been employed to reduce what is often, if somewhat misleadingly, referred to as a motion artifact. Motion is an essential part of cardiac function, of course. Electrical activation, Ca^2+^-mediated excitation-contraction coupling, contraction, relaxation and diastolic stretching, are all well-established targets for imaging. They are closely linked, over timescales from near-instantaneous beat-by-beat responses (e.g., stretch-activation of ion channels, Quinn and Kohl, 2021), over metabolic feedback to electro-mechanical function over minutes and hours (e.g., activation of K^+^-channels that open in response to a reduction in ATP Van Wagoner and Lamorgese, 1994, on to tissue remodeling over years (e.g., during aging and in disease, Lyon et al., 2020). By restricting motion for the sake of functional observation, we alter the very system we wish to study (Kappadan et al., 2020).

Increasingly, therefore, attempts are being made to image cardiac structure and function while keeping the mechanical environment as close to *in vivo* conditions as possible. For cardiac cells and cultures, this involves tools to stretch single cells (Iribe et al., 2007) or cell cultures (Zhang et al., 2008). These have given rise to otherwise “hidden” physiological responses, such as the discovery of stretch-induced Ca^2+^ release from the sarcoplasmic reticulum (Iribe et al., 2009), or the mechanistic explanation of stretch-induced conduction slowing by surface membrane integration of caveolae (Pfeiffer et al., 2014). Optical voltage mapping in actively contracting Langendorff-perfused hearts, using ratiometry and software motion correction, reveals more physiologically relevant information about the electromechanical and energetic performance of the heart (Kuzmiak-Glancy et al., 2018; Kappadan et al., 2020).

It remains more difficult to take “motion” as an input parameter to ultrastructural research. Electron microscopy, for example, is conducted on fixed samples—so motion as such is excluded. Efforts have been made to control the mechanical state of cells or tissue, prior to fixation, and to use sarcomere length as a qualifier of the mechanical state of CM when exploring ultrastructural cell properties (Kohl et al., 2003). However, chemical tissue fixation is slow compared to beat-by-beat dynamics. This can be addressed by high pressure freezing, where recent commercial systems have built-in optical and electrical stimulation capacity so that CM may be preserved for electron microscopy at predefined states in their mechanical cycle. This type of approach has shown, for example, that the T-tubular system of ventricular CM (see section 2/Figure 2) is “squeezed” twice on every heartbeat (during diastolic stretch and during systolic contraction), and that this gives rise to an advection-mediated component of T-tubular content exchange (Rog-Zielinska et al., 2021). Over the next decade this direction of work should allow examination of the cross-talk between cardiac electrics, mechanics, metabolics, (epi-)genetics and structure in a time-resolved spatial manner as part of basic cardiac research.

All of these developments will also benefit from improved algorithms for “motion tracking”—i.e., computational post-processing that allows one to maintain the association between parameters recorded on fixed detectors (from optical mapping systems to echo and magnetic resonance imaging) with the underlying moving signal source (Inagaki et al., 2004). This can increasingly be done in a fiducial marker-free manner, using optical mapping data (Christoph and Luther, 2018), or even a combination of optical and echo data gathering (Christoph et al., 2018). The latter paper superbly illustrates the interrelation of electrical and mechanical waves, highlighting their close association in 3D myocardium.

In the clinical setting, tracking of cardiac motion not only provides important information of the ultimate function of the heart - its ability to pump blood - but it conveys insight into regional differences of activation and relaxation of ventricular myocardium. “Dys-synchrony” is problematic for cardiac function, and mechanical mapping may provide insight into spatial heterogeneities, even of underlying electrical properties. In long QT syndrome, for example, disease diagnosis can be improved if mechanical properties are tracked (Lang et al., 2016). The benefit here is that it is comparatively straightforward to track motion non-invasively in 3D, and certainly easier to do so than to track electrical activity in 3D within the whole heart. A remaining caveat is that structural imaging to discern motion only offers direct insight into strain amplitudes and dynamics: thus far, there is no non-invasive technique to measure stress within the myocardium. In basic science, fluorescent reporters of stress are being developed (Meng et al., 2008; Grashoff et al., 2010; Meng and Sachs, 2012) which may pave the way toward—at least experimentally—tracking of both strain and stress in the heart. Metabolic optical imaging using native tissue fluorophores, e.g., NADH, is yet another clinically-translatable optical approach to quantifying cardiac function in health and disease with high spatial resolution (Wengrowski et al., 2014).

An exciting current development in dynamic cardiac 3D imaging is photoacoustic (or optoacoustic) imaging (Lin et al., 2018; Karlas et al., 2019). This uses a pulsed laser to deliver light into tissue. There, photons interact with suitable reporters, causing their transient thermoelastic expansion. The ultrasonic waves generated by this can be detected using ultrasonic transducers and analyzed to produce images of structure and function. Reporters can be native molecules, such as hemoglobin (whose photo-acoustic signature changes with oxygenation—for example allowing one to detect cancer in patients, Zalev et al., 2019), transgenically expressed probes such as GCaMP in experimental animals (Gottschalk et al., 2019), or dyes such as indocyanine green (Deán-Ben et al., 2015). The latter approach has been used for tomographic imaging of the beating, isolated mouse heart *in situ*. If this technique could be used in combination with recently developed photoacoustic voltage-sensitive dyes that allow one to “listen to voltage” (Zhang et al., 2017). This would represent a break-through for measuring transmural electro-mechanical dynamics in the heart.

## 6. Conclusions

This article highlights recent developments in the technical areas of optical voltage measurements and optogenetic applications to cardiac electrophysiology. While similar techniques are being developed in neuroscience, cardiac tissue presents a series of additional challenges related to the highly scattering nature of the myocardium and movement associated with contraction. In the past, progress in electrophysiology has relied on developments in physics and engineering. This article describes some of the fundamentals of physics that underlie optical techniques used to measure and manipulate the electrical activity of the heart. The prospect of translation of these research techniques to the clinic is also discussed as is their recent use in the industrial setting of toxicology screening for adverse effects on cardiac electrophysiology. In all of these situations, the use of optical tools to measure and manipulate cardiac electrophysiology is a rapidly advancing topic that promises to yield new insights and techniques to address the treatment of cardiac arrhythmias.

## Author Contributions

MCM, PK, LS, and GS planned the content. MCM and GS organized the writing. AK edited the initial submission. All authors contributed to the article and approved the submitted version.

## Conflict of Interest

CA is an owner and employee of Potentiometric Probes, which develops and sells voltage-sensitive dyes. GS is a non-salaried, founder, executive and Chief Scientific Offer of Clyde Biosciences Ltd (UK). The remaining authors declare that the research was conducted in the absence of any commercial or financial relationships that could be construed as a potential conflict of interest.

## Publisher's Note

All claims expressed in this article are solely those of the authors and do not necessarily represent those of their affiliated organizations, or those of the publisher, the editors and the reviewers. Any product that may be evaluated in this article, or claim that may be made by its manufacturer, is not guaranteed or endorsed by the publisher.
